# Epigenetic Regulation of *ZNF687* by miR-142a-3p and DNA Methylation During Osteoblast Differentiation and Mice Bone Development and Aging

**DOI:** 10.3390/ijms26052069

**Published:** 2025-02-27

**Authors:** Débora Varela, Tatiana Varela, Natércia Conceição, M. Leonor Cancela

**Affiliations:** 1 Centre of Marine Sciences, University of Algarve, 8005-139 Faro, Portugal; a44771@ualg.pt (D.V.); a44770@ualg.pt (T.V.); 2 Faculty of Medicine and Biomedical Sciences, University of Algarve, 8005-139 Faro, Portugal; 3 Algarve Biomedical Center, University of Algarve, 8005-139 Faro, Portugal

**Keywords:** osteoblast differentiation, *ZNF687*, epigenetics, miRNAs, DNA methylation, bone

## Abstract

Zinc finger protein 687 (ZNF687), a transcription factor implicated in osteoblast/osteoclast differentiation and linked to Paget’s disease of bone, has unclear mechanisms in bone metabolism. Epigenetic disruptions can affect bone cell activity and contribute to bone-related diseases. This work aimed to elucidate the regulatory role of epigenetics in modulating *Zfp687* expression throughout osteoblast differentiation and bone growth/aging in mice. Differentiation of the mouse-derived osteoblast precursor cell line (MC3T3-E1) showed increased expression of osteogenic markers and decreased *Zfp687* expression. In the hindlimb bones of C57BL/6J mice, the expression of most bone-forming genes decreased from youth to adulthood, while *Zfp687* and *Runx2* expression was maintained, being only significantly reduced in old mice in comparison to young mice. Bisulfite sequencing revealed hypomethylation of the *Zfp687* promoter during MC3T3-E1 differentiation and bone growth/aging. Bioinformatics predicted miR-142a-3p, miR-122b-5p, and miR-124-3p binding sites in *Zfp687* 3′UTR, and RT-qPCR analysis showed higher expression of these miRNAs in mature osteoblasts. Transfection of a miR-142-3p mimic reduced luciferase activity in the wildtype *Zfp687* 3′UTR but not the mutant 3′UTR and downregulated the *Zfp687* gene and protein levels. In conclusion, miR-142a-3p directly targets the *Zfp687* 3′UTR, promoting its downregulation during osteoblastogenesis. Furthermore, DNA methylation does not appear to regulate *Zfp687* during osteoblast differentiation or bone development in mice.

## 1. Introduction

Bone is a mineralized connective tissue that forms the endoskeleton to provide support to the body structure and integrity of vertebrates [1]. Despite its seemingly inactive appearance, bone is metabolically dynamic and endures constant remodeling (old or damaged bone is restored by new bone) [2,3,4]. This highly complex and dynamic process is mainly regulated by two types of cells: osteoblasts, which are the bone-forming cells, and osteoclasts, which are the bone-resorbing cells [1,5]. Through a multistep differentiation pathway, mesenchymal stem cells (MSCs) differentiate into osteoprogenitors, which then progress into pre-osteoblasts and ultimately mature osteoblasts capable of secreting the bone matrix [2,6]. Osteoclasts are cells with multiple nuclei that differentiate from the monocyte/macrophage lineage of hematopoietic stem cells through the fusion of mononuclear precursors [1,3].

The balance between bone resorption and deposition is vital for effective bone remodeling and must be closely linked, not only quantitatively but also in terms of timing and spatial coordination [3,7]. Disruptions in these processes may cause several bone diseases, including osteoporosis, osteopetrosis, and Paget’s disease of bone (PDB), highlighting the importance of their precise regulation in bone health and disease [2,8].

Paget’s disease of bone is a chronic metabolic condition in which an excessive amount of osteoclast-mediated resorption is followed by accelerated and disordered bone production by osteoblasts, resulting in larger and deformed bones [9,10]. Although its etiology is still not well-comprehended, PDB exhibits a clear genetic component [10] since several genes have been associated with the disease, including *SQSTM1*, *TNFRSF11A*, *OPTN*, and *CSF1* [9,10,11,12]. In addition, variants in the zinc finger protein 687 (*ZNF687*) gene are linked to more severe PDB (earlier onset and an increased number of affected bones), occasionally accompanied by giant cellular tumors [13]. *ZNF687* encodes a C2H2 zinc finger protein with a potential DNA-binding domain that is present in bone and in many other tissues, and [13,14] it shares approximately 80% nucleotide similarity with its mouse ortholog, the *Zfp687* gene [15]. Although it is well-recognized that certain C2H2 zinc finger proteins play an important role in regulating the growth and maintenance of the skeleton, little is known about ZNF687’s precise function and regulation in bone metabolism [16]. Overexpression of *ZNF687* has also been observed in osteoblasts differentiated from molar follicles and throughout zebrafish caudal fin regeneration, suggesting a role for ZNF687 in the proliferation and differentiation of bone cells [13]. It has also been shown that *ZNF687* is highly expressed during human and murine osteoclast development, highlighting its key role in driving osteoclast differentiation [15].

As we have demonstrated in our previous study, several bone-related transcription factors regulate *ZNF687* expression, with this regulation being influenced by DNA methylation [17]. Relevant progress is being made in epigenetic research on bone cells, providing evidence for changes in non-coding RNAs and in histone and DNA methylation patterns as cells progress toward differentiation and maturity, thus suggesting an important role in bone remodeling [18]. DNA methylation involves the incorporation of a methyl group into the cytosine base at a CpG site by DNA methyltransferases (DNMTs). This DNA modification can regulate gene transcription by preventing the binding of transcription factors or by promoting the binding of proteins with an affinity for methylated DNA [19]. miRNAs are short (21–25 nucleotides long) single-stranded RNA molecules that function as gene expression regulators [20]. Consequently, a deeper knowledge of the epigenetic regulation of PDB-associated genes, such as *ZNF687*, in bone cells is essential for uncovering the mechanisms underlying this disease. Despite its potential relevance for early diagnosis and the development of novel therapeutic strategies, the specific regulatory pathways of *ZNF687* during osteoblast differentiation and bone development remain poorly characterized. This study aims to fill this gap by investigating *Zfp687* expression and its epigenetic regulation during the differentiation of the pre-osteoblastic MC3T3-E1 cell line, as well as throughout the development and aging of murine long bones. By elucidating how *Zfp687* is regulated, this research could provide critical insights into its role in bone physiology and disease.

## 2. Results

### 2.1. Mineralization and Expression of Zfp687 and Osteogenic Marker Genes Throughout MC3T3-E1 Osteoblast Differentiation

**Figure 1:**
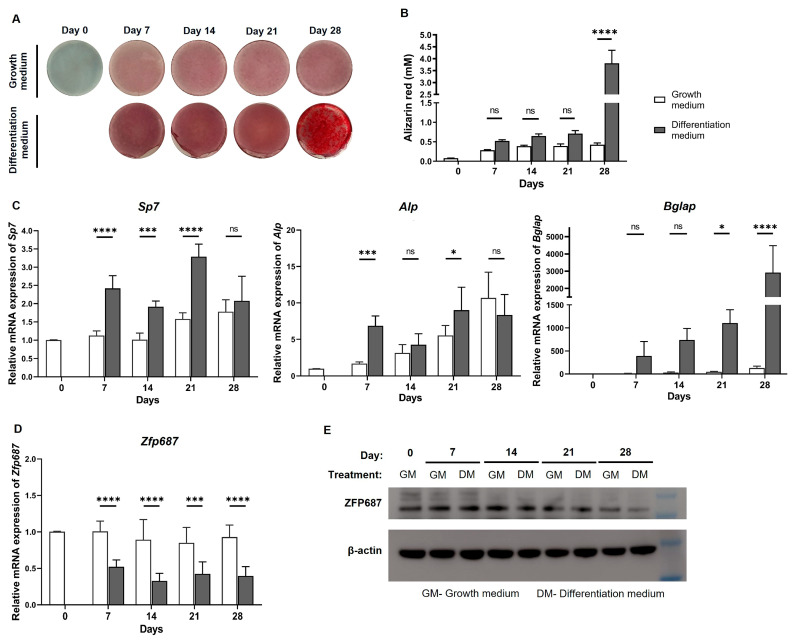
Osteoblast differentiation of MC3T3-E1 cells under exposure to osteogenic differentiation media for 28 days: Mineralization, osteogenic markers, and *Zfp687* expression. (**A**) Alizarin red S staining for mineralized nodules. (**B**) Quantification of calcium-bound alizarin red S by densitometry analysis at 550 nm. (**C**) Relative mRNA expression of *Sp7*, *Alp*, and *Bglap* at various time points during the differentiation. (**D**) Relative mRNA expression of *Zfp687*. (**E**) ZFP687 protein expression determined by Western blot. GM: growth medium; DM: differentiation medium. All graphical data represent the mean ± standard deviation (SD) of duplicates from three independent experiments (*n* = 6). Two-way ANOVA followed by Sidak’s multiple comparisons was used for statistical analysis, with significance determined at * *p* < 0.05; *** *p* < 0.001; and **** *p* < 0.0001, and ns indicating no statistical difference.

One indicator of the differentiation of osteoblasts into mature osteoblasts is the mineralization of the extracellular matrix. Mineralization results from the deposition of hydroxyapatite mineral crystals composed mainly of calcium and phosphorus (secreted by mature osteoblasts) in the extracellular matrix [21]. Alizarin red S staining is a standard method widely used as an indicator of whether the differentiation and consequent mineralization of osteoblasts has occurred [22]. Although it does not specifically detect hydroxyapatite, alizarin red S binds to calcium, a main constituent of hydroxyapatite. Therefore, we evaluated the mineralization of MC3T3-E1 cells throughout the 28 days of osteogenic treatment using alizarin red S staining. MC3T3-E1 cultured in the presence of an osteogenic cocktail showed stronger red staining in comparison to the control cells cultured in growth medium alone. This was more noticeable at 28 days of differentiation, as more mineralized nodules were observed (Figure 1A), which was confirmed by the quantification of the mineralized matrix (Figure 1B). Therefore, alizarin red S staining confirmed the differentiated phenotype of the cells treated with the osteogenic differentiation medium.

The differentiation of osteoblasts is accompanied by alterations in the mRNA levels of bone markers [23,24]. To characterize the gene expression profile of MC3T3-E1 cells across the known phases of osteoblast differentiation [25], and to further confirm that cells were differentiating into mature osteoblasts, the expression levels of several bone markers were analyzed (Figure 1C). The expression of osterix/*Sp7* (marker of committed osteoblasts [26,27]) and alkaline phosphatase/*Alp* (earlier osteoblast differentiation marker [28]) significantly increased during the early stage of osteoblastogenesis (on day 7) and was sustained throughout the entire differentiation process. The expression of osteocalcin/*Bglap* (matrix protein produced by mature osteoblasts, and a later differentiation marker [29]) also significantly increased, with a peak observed on day 28. Overall, our results demonstrated higher expression levels of these genes in osteogenic-treated cells in comparison to control cells, confirming the occurrence of osteogenic differentiation (Figure 1C).

To study the potential role of ZFP687 in osteoblastogenesis, the expression levels of both *Zfp687* mRNA and protein were analyzed throughout the differentiation of pre-osteoblastic MC3T3-E1 cells. Our results show that *Zfp687* expression was significantly lower in differentiating osteoblasts compared to their undifferentiated precursors throughout the 28 days of osteogenic treatment (Figure 1D). Western blot analysis also showed a decrease in ZFP687 protein expression in differentiating osteoblasts, but this was only observed on day 28 of osteogenic treatment (Figure 1E). Inconsistencies between mRNA and protein levels have been frequently reported in the literature, with possible explanations including post-transcriptional regulation, translation, and turnover rates [30,31].

### 2.2. Expression of Zfp687 and Bone Marker Genes Throughout Bone Growth/Development

**Figure 2:**
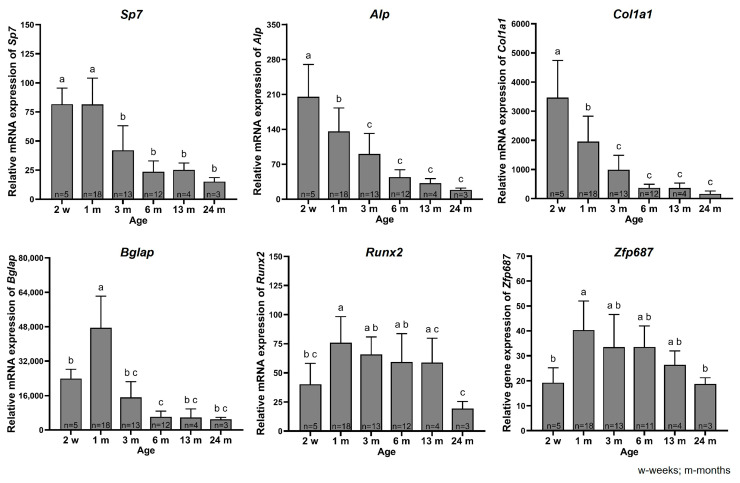
Relative mRNA expression of bone markers in hindlimb long bones of C57BL/6J mice at different ages. The relative mRNA expression of *Sp7*, *Alp, Col1a1, Bglap, Runx2, and Zfp687* was measured at neonatal (2 weeks), young (1 month), adult (3 months and 6 months), middle-age (13 months), and old (24 months) stages. Data are presented as the mean relative mRNA expression ± SD. One-way ANOVA followed by Tukey’s multiple comparisons was used for statistical analysis. Significant differences are indicated by distinct lowercase letters. w—weeks; m—months.

To determine whether the expression of *Zfp687* changes with the growth, maturation, and aging of bone, we evaluated the expression of *Zfp687* and bone formation genes in the hindlimb long bones of neonatal (2-week-old), young (1 month), adult (3 months and 6 months), middle-aged (13 months), and old (24 months) C57BL/6J mice (Figure 2). From 2 weeks to 1 month, the expression levels of *Alp* and collagen type I alpha 1 chains (*Col1a1*) in bone decreased significantly while the expression of *Bglap*, runt-related transcription factor 2 (*Runx2*), and *Zfp687* significantly increased. There were no significant differences in osterix (*Sp7*) expression between these two developmental stages. The expression of *Sp7*, *Alp*, *Col1a1*, and *Bglap* in bone was significantly reduced by 1.9-fold, 1.4-fold, 2-fold, and 3.1-fold, respectively, from 1 to 3 months of age. Although the expression of these four genes further declined between 3 and 6 months of age, the changes were not statistically significant. No further alterations in gene expression were observed between 6 and 24 months of age (Figure 2).

The early osteoblast transcription factors *Runx2* and *Zfp687* share a very similar expression pattern in mouse bone development. Although their expression did not alter between 1- and 13-month-old mice, 24-month-old mice showed significantly lower mRNA levels in comparison to 1-month-old mice (3.9-fold and 2.1-fold decreases for *Runx2* and *Zfp687*, respectively) (Figure 2).

### 2.3. CpG Sites in the Zfp687 Promoter

**Figure 3:**
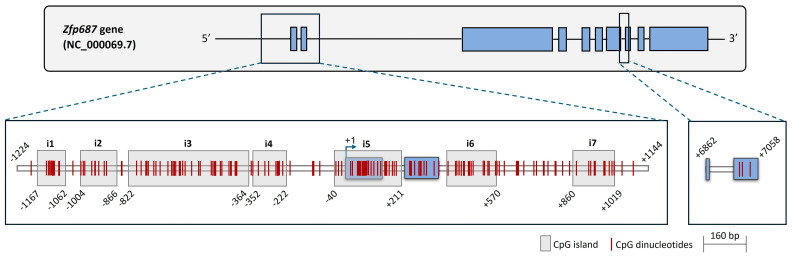
Bioinformatics analysis of CpG dinucleotides in mouse *Zfp687*. Schematic representation of CpG islands and CpG sites in *Zfp687*, spanning from −1224 to +1144 and from +6862 to +7058 relative to the transcription start site (+1). CpG islands and CpG dinucleotides are marked by light grey boxes and vertical red lines, respectively. Start/end positions are indicated in relation to the transcription start site. *Zfp687* exons and introns are represented by blue boxes and horizontal lines, respectively.

The mouse *Zfp687* gene consists of ten exons and nine introns and produces two transcripts, according to the annotated nucleotide sequences from the NCBI database (Figure S1A). The gene structure of *Zfp687* (exon–intron–exon) is highly similar to that of the human gene, although the human gene contains one additional exon present only in transcript variant 1. Analysis using the ConSuf tool showed that *Zfp687* is conserved across species, with conservation scores for each nucleotide position shown in Figure S1B. We then analyzed the presence of CpG islands and CpG dinucleotides in the *Zfp687* promoter region, spanning from −1224 to +1144 relative to the transcription start site (TSS). This region is rich in CpG islands, with seven predicted CpG islands (Figure 3), and contains 154 identified CpG dinucleotides. Nucleotide alignment of the mouse −1224/+1144 region with the corresponding human *ZNF687* region showed a high degree of conservation, including 67 conserved CpGs between mice and humans (Figure S2). A detailed description of the CpG dinucleotide locations in the analyzed *Zfp687* regions, along with the corresponding numbering, is provided in Figure S3. Together, the results suggest that the analyzed promoter region may play a functional role in gene regulation, possibly through DNA methylation. Furthermore, we also analyzed the region spanning from +6862 to +7058, which contains no CpG islands and only three CpG dinucleotides (Figures S2 and S3).

### 2.4. Methylation Status of Zfp687 During Osteoblastogenesis

To investigate the potential mechanism of regulation responsible for reduced *Zfp687* expression during osteoblast differentiation, we examined the methylation profile of selected CpG sites within the *Zfp687* promoter region (−1224/+1144) and 3′ region (+6862/+7058). Twenty-nine CpGs (numbered 23, 24, 100–108, and 130–147) within these regions were not analyzed due to technical difficulties, as we could not amplify them (absence of suitable primers). Since Z*fp*687 downregulation was more pronounced at 14 days of MC3T3-E1 differentiation, we selected this time point for the methylation analysis and compared it to the levels present in untreated cells (day 0).

**Figure 4:**
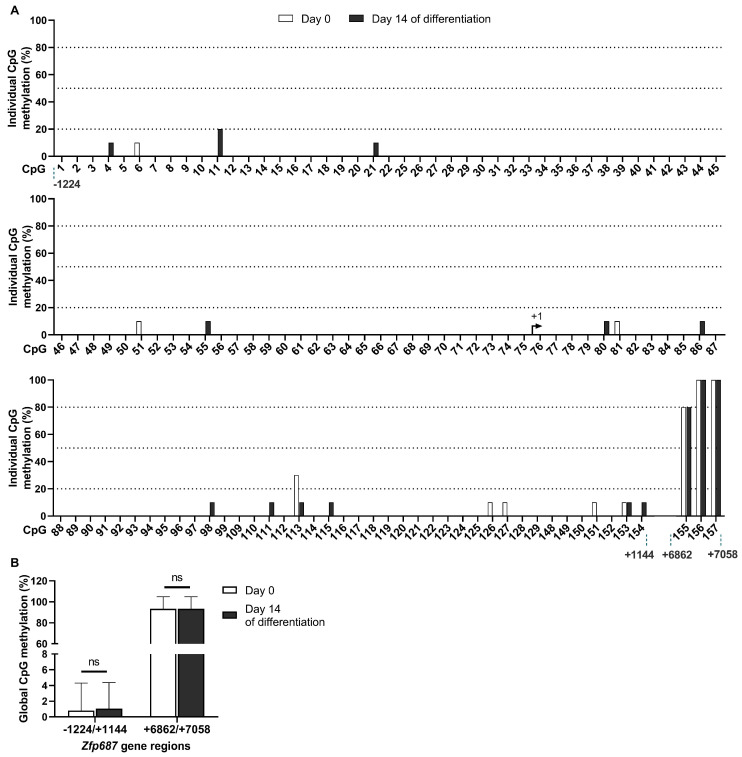
CpG methylation of *Zfp687* during osteoblastogenesis. (**A**) Methylation levels of individual CpG sites in the *Zfp687* gene in MCET3-E1 pre-osteoblasts (day 0) and differentiating osteoblasts (day 14). Bars represent the percentage of methylation for each CpG site, numbered from 1 to 157, calculated from ten individual clones. (**B**) Global CpG methylation within the *Zfp687* regions −1224/+1144 and +6862/+7058 at day 0 and day 14 of MC3T3-E1 differentiation. Bars represent the mean methylation (%) ± SD of all CpGs within each region. Statistical analysis was performed using the Mann–Whitney U test. ns indicates no statistical difference.

Our results show that, in both differentiated osteoblasts and undifferentiated MC3T3-E1 cells, 128 CpGs (CpG numbers 1–22, 25–99, 109–129, and 148–154) are hypomethylated (≤30% methylation), while three CpGs (CpG number 155 to 157) are highly methylated (≥80% methylation). On day 0 and day 14 of differentiation, 117 and 113 CpGs, respectively, were completely unmethylated (Figure 4A). Interestingly, the hypomethylated region corresponds to the *Zfp687* promoter, while the hypermethylated region corresponds to the 3′ region. These data align with the concept that genomic DNA areas characterized by low CpG site density are typically hypermethylated, likely exerting minimal influence on transcriptional activation status [32].

We found no differences in the *Zfp687* methylation levels between the two analyzed conditions. The overall CpG methylation of the *Zfp687* promoter region and 3′ region is approximately 1% and 93%, respectively, at both day 0 and day 14 of differentiation (Figure 4B). These data indicate that the promoter region of *Zfp687* is hypomethylated, regardless of their differentiation status. Therefore, the downregulation of *Zfp687* in osteoblastogenesis may not be caused by methylation of the analyzed regions.

### 2.5. Methylation Status of Zfp687 Throughout Bone Growth/Development

**Figure 5:**
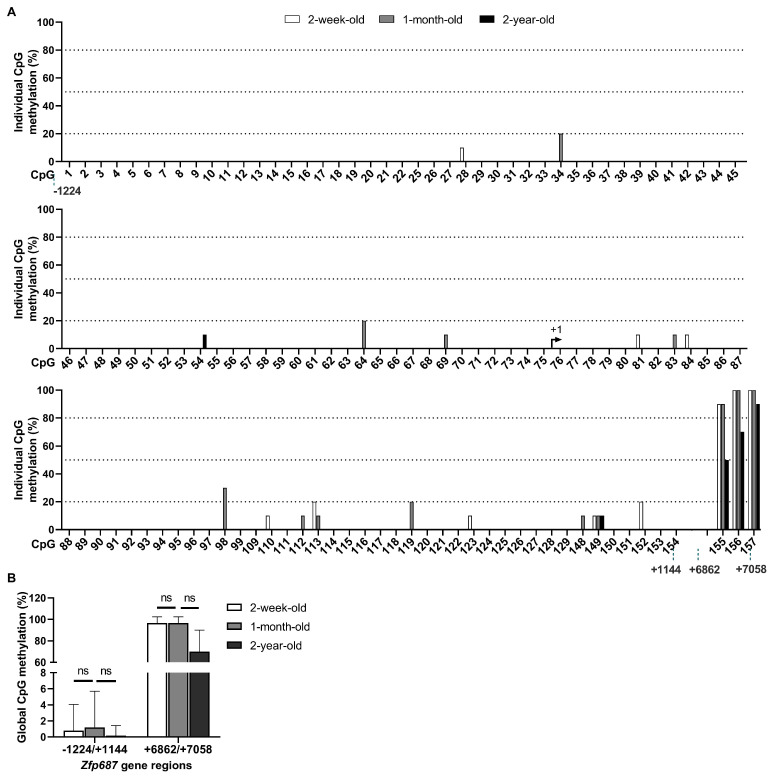
CpG methylation of *Zfp687* in hindlimb long bones from mice at different ages. (**A**) Methylation levels of individual CpG sites in the *Zfp687* gene in mice at 2 weeks, 1 month, and 2 years of age. Bars represent the percentage of methylation for each CpG site, numbered from 1 to 157, calculated from ten individual clones. (**B**) Global CpG methylation within the *Zfp687* regions −1224/+1144 and +6862/+7058 in mice at 2 weeks, 1 month, and 2 years of age. Bars represent the mean methylation (%) ± SD of all CpGs within each region. Statistical analysis was performed using the Kruskal–Wallis test. ns indicates no statistical difference.

To determine whether *Zfp687* expression in the bone of mice at different developmental stages is regulated by DNA methylation, CpG methylation levels were examined in the hindlimb long bones of wildtype mice, ranging from the active growth phase in newborns and young mice to the diminished growth and increased bone loss observed in 2-year-old mice. The results obtained for the methylation profile of CpG sites within the *Zfp687* promoter region (−1224/+1144) and 3′ region (+6862/+7058) are shown in Figure 5. Our results show that, regardless of bone developmental stage, 128 CpGs (CpG numbers 1–22, 25–99, 109–129, and 148–154) are hypomethylated (methylation ≤ 30%), while the three CpGs (CpG numbers 155 to 157) located in the 3′ region are highly methylated. At 2 weeks old, 1 month old, and 2 years old, 117, 115, and 123 CpGs, respectively, are completely unmethylated (Figure 5A). We found no statistical differences in the *Zfp687* global methylation levels between the three groups (Figure 5B).

### 2.6. In Silico Characterization of the Zfp687 3′UTR Regulatory Region and MicroRNA Expression in MC3T3-E1 Osteoblast Differentiation

**Figure 6:**
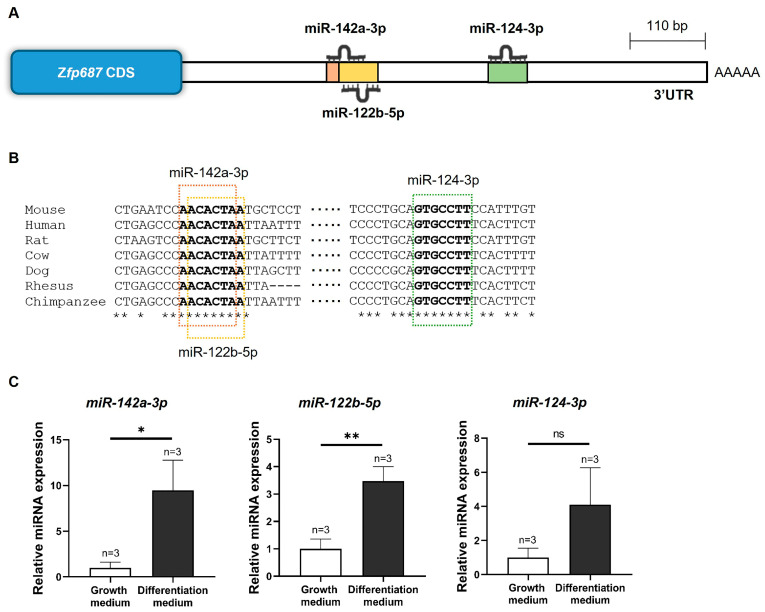
Prediction of miRNA binding sites in the 3′UTR of mouse *Zfp687* and miRNA expression during MC3T3-E1 differentiation. (**A**) Schematic representation of potential miRNA binding sites in the 3′UTR of mouse *Zfp687*. (**B**) Alignment of miR-142a-3p, miR-124-3p, and miR-122b-5p binding sequences across vertebrates. Sequences in bold highlight the putative miRNA binding sites within the 3′UTR and asterisks (*) denote nucleotide positions that are 100% conserved. (**C**) Relative expression of miRNAs in MC3T3-E1 pre-osteoblasts (cultured in growth medium) and differentiating osteoblasts (cultured in differentiation medium for 14 days), normalized to U6 small RNA. Data are presented as the mean relative expression ± SD from three independent experiments (*n* = 3). The unpaired *t*-test was used for statistical analysis, with significance determined at * *p* < 0.05; ** *p* < 0.01. ns indicates no statistical difference.

We investigated the possibility that *Zfp687* might be regulated post-transcriptionally by microRNAs (miRNAs). Using bioinformatic tools (TargetScan, miRDB, and STarMir), we identified miR-142a-3p, miR-124-3p, and miR-122b-5p as miRNAs that could potentially bind to the 3′UTR of *Zfp687*. Figure 6A displays the location of these miRNA binding sites within the mouse *Zfp687* 3′UTR, while Figure 6B shows their conservation. The binding sites for miR-142a-3p and miR-122b-5p were highly conserved across more than 50 mammalian species, while miR-124-3p showed conservation in over 60 mammalian species and five bird species. According to this analysis, the identified binding sites appeared to have been highly conserved throughout evolution.

To investigate whether miR-124-3p, miR-142a-3p, and miR-122b-5p are involved in the regulation of osteoblastogenesis, their expression levels were examined in differentiating MC3T3-E1 cells. As shown in Figure 6C, miR-142a-3p and miR-122b-5p were significantly upregulated, with increases of approximately 10-fold and 4-fold, respectively, in MC3T3-E1 after 14 days of treatment with an osteogenic medium. These results suggest that miR-142a-3p and miR-122b-5p might positively regulate osteoblast differentiation and extracellular matrix mineralization.

### 2.7. Zfp687 Regulation by miR-142a-3p

**Figure 7:**
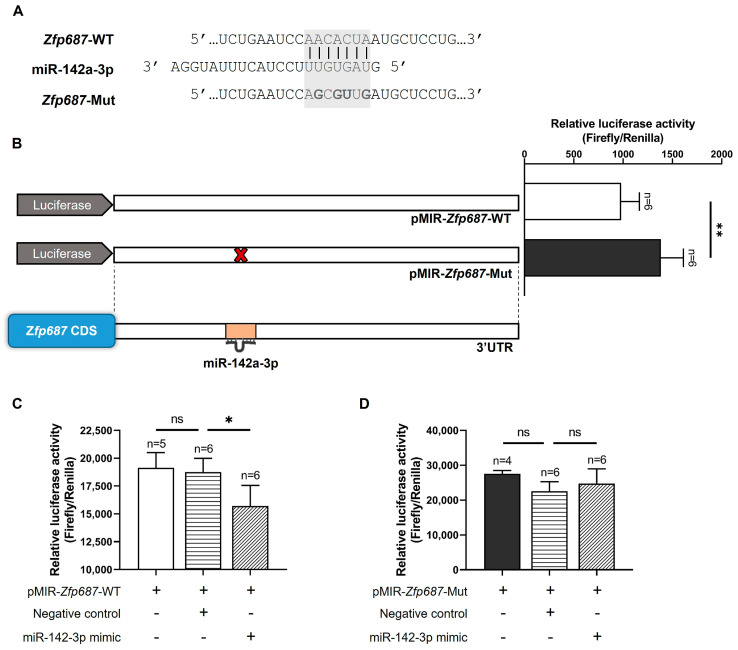
Functional analysis of miR-142a-3p binding in the *Zfp687* 3′UTR. (**A**) Nucleotide sequences of wildtype (WT) and site-directed mutated (Mut) binding sites for miR-142a-3p in the *Zfp687* 3′UTR. The seed sequence is highlighted in a grey shaded box. Mutations in the miR-142a-3p binding site of the 3′UTR are indicated in bold. (**B**) Relative luciferase activity of the WT (pMIR-*Zfp687*-WT) and mutated *Zfp687* (pMIR-*Zfp687*-Mut) in MC3T3-E1 cells. Data are presented as the mean relative luciferase activity (ratio between Firefly luciferase of the 3′UTR construct and the Renilla luciferase of the internal control) ± SD. Statistical analysis was performed using the unpaired *t*-test with Welch’s correction, with significance determined at ** *p* < 0.01. (**C**,**D**) Relative luciferase activity of the WT or mutated *Zfp687* in HEK-293 cells co-transfected with the miR-142-3p mimic or negative control. One-way ANOVA followed by Tukey’s multiple comparisons was used for statistical analysis, with significance determined at * *p* < 0.05. ns indicates no statistical difference.

To further investigate the molecular mechanisms regulating *Zfp687* during osteoblast differentiation, we focused our study on miR-142a-3p for two key reasons. First, among those identified in silico, it emerged as the most significantly upregulated miRNA in differentiating osteoblasts. Second, previous studies have demonstrated its overexpression during the osteoblast differentiation of hFOB1.19 cells, suggesting an essential role in promoting osteogenic processes [33]. The 3′UTR of the *Zfp687* gene containing one miR-142a-3p binding site was cloned into the pMIR-report vector downstream of a luciferase reporter. Additionally, a second luciferase reporter construct was generated in which the *Zfp687* 3′UTR contained a mutated sequence at the miR-142a-3p binding site (Figure 7A). These constructs were transiently transfected into MC3T3-E1 cells, which we had previously shown to endogenously express miR-142a-3p. Our results show a significant increase in luciferase activity when the miR-142a-3p binding site is mutated (Figure 7B). Then, both wildtype and mutated *Zfp687* constructs were co-transfected with either a miR-142-3p mimic or a negative control into HEK-293 cells. Our results show that the miR-142-3p mimic significantly reduced the luciferase activity of the wildtype *Zfp687* 3′UTR in comparison with the negative control (Figure 7C). In contrast, the luciferase activity of the mutated *Zfp687* 3′UTR was unaffected by the miR-142-3p mimic (Figure 7D). Therefore, mutating four nucleotides within the miR-142a-3p binding site completely eliminated this repressive effect, suggesting that *Zfp687* is a direct target of miR-142a-3p and that these nucleotides are essential for miR-142-3p-mediated regulation.

**Figure 8:**
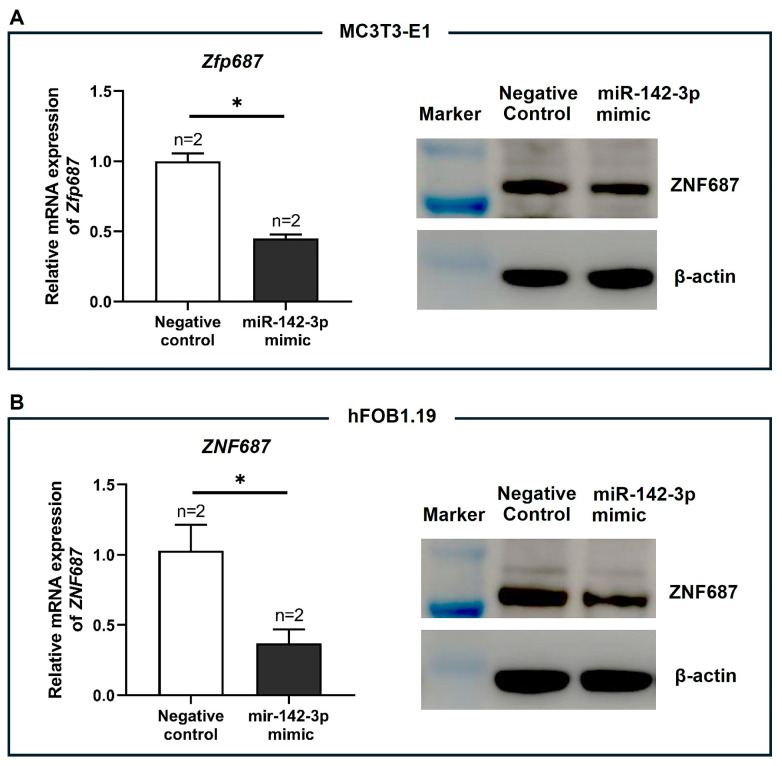
Effect of miR-142-3p on *Zfp687*/*ZNF687* expression. Relative *Zfp687*/*ZNF687* mRNA expression and protein levels in (**A**) MC3T3-E1 and (**B**) hFOB1.19 cells upon transfection of the miR-142-3p mimic or negative control. Bars represent the mean relative expression ± SD from two independent experiments (*n* = 2). An unpaired *t*-test was used for statistical analysis, with significance determined at * *p* < 0.05.

We then transfected MC3T3-E1 and hFOB1.19 cells with either the miR-142a-3p mimic or a negative control. As shown in Figure 8, overexpression of miR-142-3p significantly decreased *Zfp687*/*ZNF687* mRNA levels and reduced endogenous Zfp687/ZNF687 protein levels in both MC3T3-E1 and hFOB1.19 cell lines. These results indicate that miR-142a-3p downregulates *ZFP687*/*ZNF687* expression through mRNA degradation and translational inhibition.

## 3. Discussion

The processes of osteoblast differentiation and bone formation are controlled by a complex network of extracellular molecules, including transcriptional factors [34,35,36,37]. These factors work together to influence osteoblast lineage commitment, maturation, and mineralization, ultimately coordinating bone growth, remodeling, and repair [35]. *ZNF687* encodes a transcription factor involved in bone remodeling regulation and is associated with PDB [13,38]. However, the exact role of ZNF687 in bone formation remains unclear, and its regulatory mechanism in bone cells has not been fully explored. In this work, we focus on examining the expression profile of *Zfp687* and its epigenetic regulation throughout osteoblast differentiation in MC3T3-E1 cells and in the hindlimb bones of mice at various developmental, growth, and aging stages. To the best of our knowledge, this is the first work to explore the epigenetic regulation of *Zfp687* in bone cells, with particular emphasis on the methylation profile of *Zfp687* during osteoblastogenesis and bone growth/aging, as well as on the regulation of *Zfp687* by miRNAs.

The MC3T3-E1 cell line has been extensively used to study osteoblast differentiation and function due to its ability to undergo the osteoblastogenesis pathway when cultured in a medium supplemented with β-glycerophosphate and ascorbic acid [23]. These cells exhibit a clear transition through distinct stages, from proliferative pre-osteoblasts to mature osteoblasts capable of synthesizing and mineralizing the extracellular bone matrix, resembling the differentiation process and behavior of primary osteoblasts even if, being an in vitro model, they do not fully replicate the complexity of osteoprogenitor cells in vivo. Nevertheless, MC3T3-E1 cells express osteogenic markers in a time-dependent manner, allowing for the accurate monitoring of each differentiation stage and confirming their usefulness in bone biology research. Three different phases of osteoblast differentiation are reported: proliferation (3 to 7 days after cells are confluent); extracellular matrix accumulation; and terminal differentiation, evidenced by mineralization [25,39]. In our experiments, the detection of a mineralized bone matrix and the significant upregulation of osteoblast markers (*Sp7*, *Alpl*, *Col1a1*, and *Bglap*) in MC3T3-E1 cells upon osteogenic treatment provide evidence of successful differentiation.

Our study showed that *Zfp687* expression remains downregulated throughout the 28-day period of MC3T3-E1 osteoblast differentiation. In contrast, Gianfrancesco et al. reported upregulation of *Zfp687* expression during osteoblast differentiation in mouse bone marrow-derived mesenchymal stem cells (BMSCs) [15] and in human molar follicles [13]. One possible reason for this discrepancy lies in the initial differentiation state of the cells used. While MC3T3-E1 cells are committed pre-osteoblasts, already progressing along the osteoblast lineage, BMSCs and molar follicles are multipotent cells, suggesting that ZNF687 may be more required for the initial commitment to the osteoblast lineage. Altogether, these data indicate that *Zfp687* expression may be regulated differently depending on the cellular origin and differentiation stage of the osteogenic lineage and suggest that ZFP687 might have a more crucial role in the early differentiation stages, rather than in terminal osteoblast maturation.

To further understand the correlation between the expression of *Zfp687* and established osteoblastogenesis markers [40] during normal mouse development, we investigated the mRNA levels of both *Zfp687* and a set of osteoblast- and extracellular matrix-related genes in hindlimb long bones from neonatal to aged C57Bl/6 mice. The expression of earlier osteoblastogenesis markers, *Alp* and *Col1a1*, significantly peaks at the neonatal stage and decreases significantly in youth, while the expression of known markers of bone maturation, such as *Bglap*, significantly increases, indicating a shift toward more advanced osteoblast activity and bone mineralization. Expression of *Sp7*, *Alp*, *Col1a1*, and *Bglap* was significantly decreased from young (1-month-old) to adult (3-month-old) mice, and as aging progressed, these markers showed only modest, non-significant trends of decline, reflecting a shift toward maintaining bone homeostasis rather than active growth. Similarly, others have also shown a decrease in the expression of bone formation genes as age progressed. Silva et al. showed decreased expression of *Sp7*, *Alp*, *Col1a1*, *Bsp*, and *Bglap* in a different mouse strain (female BALB/c) from 2 to 4 months of age [41]. Cao et al. also reported that *Alp* and *Col1a1* expression levels were lower in older C57Bl/6 male mice compared to young mice [42]. In mice, skeletal maturity is reached around 12 to 16 weeks, when the highest bone density and mechanical properties are achieved [40,43,44]. As mice age from middle to old age, bone mass and strength progressively decline [43,45]. Since our results show that gene expression levels of bone formation-related genes appear to be maintained, this could indicate that bone remodeling probably shifted towards more bone resorption. Although we did not examine the expression of bone resorption marker genes, Cao et al. showed that, in C57Bl/6 male mice, the expression of *Rankl* increased while osteoprotegerin decreased with advancing age, favoring osteoclast activity [42]. *Runx2* expression levels remained stable from youth to middle age, consistent with findings by Silva et al. in BALB/c mice [41], but decreased in 24-month-old compared to 1-month-old mice. Notably, *Zfp687* followed a similar expression trend, closely mirroring *Runx2* throughout bone development, maturation, and aging in mice. The gene *Runx2* encodes a transcription factor crucial for the initial commitment of mesenchymal stem cells to the osteoblast lineage, marking the first essential step in osteoblast differentiation and bone formation [46]. *Runx2* expression reaches its highest levels in pre-osteoblasts and declines as osteoblasts mature [34,46]. Accordingly, these data further support our hypothesis that ZFP687 is primarily required during the early stages of osteoblast differentiation. Although there are no reports on the interaction between ZFP687 and RUNX2 in the literature, as both are transcription regulators, they might directly interact to co-regulate and mediate the transcription of key genes with critical functions in osteoblast differentiation or even regulate each other’s transcriptional activity. Further investigations are needed to elucidate their relationship through methodologies such as chromatin immunoprecipitation or reporter assays. In our experiments, we included an equal number of male and female mice at each time point, as well as both cortical and trabecular hindlimb bones. Therefore, we cannot exclude the possibility of sex-specific and bone type-specific differences in *Zfp687* expression patterns. In addition, qPCR is not suitable for detecting differences in spatiotemporal expression between specific tissue regions. For that analysis, in situ hybridization and immunohistochemistry should be applied.

Epigenetic mechanisms have been previously implicated in the tight regulation of gene expression that occurs during bone formation and development. Based on evidence from the literature indicating that osteoblast marker genes (e.g., *Bglap*, *Col1a1*, and *Spp1*) change their methylation status as cells progress toward differentiation and maturity [47,48,49], we investigated the levels of *Zfp687* gene methylation in the MC3T3-E1 cell line undergoing osteoblastic differentiation and in the bones of mice at different developmental stages. Our data show that the *Zfp687* promoter is hypomethylated and the 3′ region is hypermethylated, regardless of the differentiation stage of MC3T3-E1 osteoblasts. Consistently, *Zfp687* CpG methylation in bone from mice at 2 weeks, 1 month, and 2 years of age is hypomethylated in the promoter region and hypermethylated in the 3′ region. Therefore, CpG methylation might not regulate *Zfp687* expression during these processes. However, we cannot exclude the possibility of additional regulatory regions, such as CpG sites near the promoter region, that were not analyzed, or more distal regulatory elements (e.g., enhancers or silencers) that may influence *Zfp687* expression through long-range epigenetic interactions. Functional validation studies using demethylating agents or methylation inhibitors should be performed to explore the role of distal or indirect epigenetic mechanisms.

Several reports indicate that osteoblast differentiation and bone formation are regulated by miRNAs [50,51,52]. In the present work, we found conserved predicted binding sites for miR-142a-3p and miR-122b-5p in the *Zfp687* 3′UTR, and their expression was significantly upregulated during MC3T3-E1 osteoblastic differentiation. Similarly, Hu et al. demonstrated that miR-142-3p is upregulated throughout hFOB1.19 osteoblast differentiation, being a critical regulator of the differentiation process [33]. Mature miR-142-3p is highly conserved among vertebrates, including mice and humans, indicating that it may have similar functions in both species [53]. Here, we validated the targeting of *Zfp687* by miR-142a-3p. Altogether, our findings suggest that under normal physiological conditions, miR-142-3p targets and downregulates *Zfp687* expression to promote the terminal maturation of osteoblasts. Future studies should explore the role of other potential epigenetic mechanisms in *Zfp687* expression, such as histone modifications and chromatin remodeling.

Numerous studies have demonstrated an association between the dysregulated expression of specific miRNAs and various pathologies, including bone diseases such as osteoporosis [54,55,56]. As miRNAs can be released into the bloodstream and detected in peripheral blood, they hold significant promise as diagnostic and prognostic biomarkers, offering valuable insights into disease onset and progression [57,58,59]. Therefore, it would be interesting to evaluate the levels of miRNAs, especially miR-142-3p, in serum from PDB patients since *ZNF687* is associated with the disease.

Despite the high availability of drug-based treatments for bone-associated disorders, their associated side effects give rise to the need to search for new approaches. Given its emergent role in osteoblast differentiation and bone development, ZNF687 could represent a promising target for therapeutic purposes in human bone-related disorders, characterized by defective (e.g., osteoporosis and osteopenia) or excessive bone formation (e.g., osteopetrosis and PDB). Therapies aimed at modulating *ZNF687* expression or activity and consequently enhancing or reducing osteoblast activity and bone formation could counteract disease progression. Approaches such as bone-targeted gene therapy via recombinant adeno-associated viruses (rAAVs) have demonstrated great potential for the treatment of bone disorders [60,61,62]. Therefore, rAAV-mediated expression of *ZNF687* or rAAVs carrying miR-142-3p specifically in osteoblasts might have the potential to treat these disorders.

In conclusion, our study contributed to elucidating the regulatory role of epigenetics in modulating the expression of *Zfp687*, the mouse ortholog of human *ZNF687*, during osteoblast differentiation using an in vitro approach and during bone formation, growth, and aging using an in vivo mouse model. Our findings support the hypothesis that ZFP687 is primarily required in the early phases of osteoblast differentiation. Furthermore, while our data suggest that DNA methylation may not be a regulatory mechanism for *Zfp687* expression during both osteoblastogenesis and bone growth/aging in mice, they indicate that miR-142a-3p directly targets the 3′UTR of *Zfp687*, contributing to its downregulation throughout the differentiation of MC3T3-E1 pre-osteoblasts.

## 4. Materials and Methods

### 4.1. In Silico Analysis of the ZNF687 Gene

The nucleotide sequences of the human *ZNF687* and mouse *Zfp687* annotated transcript variants were collected from the nucleotide database of the National Center for Biotechnology Information (NCBI; www.ncbi.nlm.nih.gov, accessed on 19 July 2023). The transcripts’ structures were determined by aligning the transcript sequence with *ZNF687*’s genomic sequence. The ConSurf tool was used to analyze *Zfp687* conservation across 123 species.

Mouse *Zfp687* nucleotide sequences from positions −1224 to +1144 and +6862 to +7058 relative to the transcription start site were obtained from NCBI (accession number: NC_000069.7). Putative CpG islands in the *Zfp687* genomic sequence were identified using the MethPrimer program (www.urogene.org/methprimer2/) with default parameters (CpG island >100 bp, CG > 50%, and Obs/Exp > 0.6). The 773 bp sequence of the *Zfp687* 3′UTR was retrieved from Ensembl (www.ensembl.org) and analyzed for potential miRNA binding sites using TargetScan (www.targetscan.org/), miRDB (www.mirdb.org/), and STarMir (http://sfold.wadsworth.org/cgi-bin/starmirWeb.pl). TargetScan predictions are based on evolutionary conservation, requiring a perfect alignment of at least seven nucleotides between the miRNA seed and 3′UTR sequences [63]. miRDB utilizes a machine learning model trained on validated miRNA–target interactions [64] and StarMir employs a statistical thermodynamics-based model to assess the structural accessibility of miRNA binding sites within mRNA secondary structures [65].

### 4.2. Cell Culture Maintenance

The mouse pre-osteoblast MC3T3-E1 cell line subclone 4 (ATCC number CRL-2593) was cultured in Alpha Minimum Essential Medium (α-MEM; Gibco, Grand Island, NY, USA) supplemented with 10% (*v*/*v*) fetal bovine serum (FBS; Invitrogen, Waltham, MA, USA) and 1% (*v*/*v*) penicillin (10,000 U/mL, Invitrogen) and streptomycin (10,000 µg/mL, Invitrogen). The human embryonic kidney 293 cell line (HEK-293, ATCC number CRL-1573) was maintained in Dulbecco’s modified eagle medium (DMEM, Gibco) supplemented with 10% (*v*/*v*) FBS, 1% (*v*/*v*) L-glutamine (Invitrogen), and 1% (*v*/*v*) penicillin (10,000 units/mL) and streptomycin (10,000 μg/mL). Both lines were subcultured every two to three days and kept at 37 °C in a 5% CO_2_ humidified atmosphere.

The human fetal osteoblastic cell line (hFOB1.19, ATCC number CRL-3602) was cultured in DMEM/F-12 (Gibco) supplemented with 10% (*v*/*v*) FBS, 0.3 mg/mL G418 (Geneticin, Gibco), and 1% (*v*/*v*) penicillin (10,000 units/mL) and streptomycin (10,000 μg/mL), and was kept at 34 °C in a 5% CO_2_ humidified atmosphere.

### 4.3. Osteogenic Differentiation

After plating on 12-well plates, MC3T3-E1 cells were grown as described above until 90% confluency was achieved. At this stage, the cells were treated with an osteogenic cocktail: culture media supplemented with ascorbic acid (50 μg/mL) and β-glycerophosphate (10 mM) to stimulate differentiation into mature osteoblasts and mineralization. The medium was renewed every two days for 28 days. In parallel, MC3T3-E1 cells were grown in culture medium as a negative control for differentiation. On days 0, 7, 14, 21, and 28 of differentiation, the cells were washed twice with phosphate-buffered saline (PBS), stained with alizarin red S, and RNA, genomic DNA (gDNA), and protein were extracted.

### 4.4. Alizarin Red S Staining

Mineralization was assessed by alizarin red S staining to confirm osteoblast differentiation and mineralization. Cells were fixed in 4% paraformaldehyde at 4 °C for 1 h and stained in a 40 mM alizarin red S solution (pH 4.2, Sigma-Aldrich, St. Louis, MO, USA) for 15 min under gentle agitation. After removing excess dye with distilled water, red nodules were examined using an inverted light microscope and photographed with a digital camera (VWR, Radnor, PA, USA). To quantify calcium deposits, cells were de-stained in 10% cetylpyridinium chloride for 15 min. The solution was collected, and absorbance at 565 nm was measured in a Synergy 4 microplate reader (BioTek, Santa Clara, CA, USA). A calibration curve was constructed using absorbance values from known alizarin red S concentrations.

### 4.5. Dissection of Mouse Hindlimb Long Bones

C57BL/6 mice were kept in groups of four to five per cage with ad libitum access to food and water and maintained in a temperature-controlled room on a 12 h light/dark cycle at the animal facility of the Algarve Biomedical Center Research Institute. Mice on the day of birth were designated as day 0. Hindlimb long bones (femurs, tibiae, and bone marrow) were collected from C57BL/6 mice (same number of females and males) aged 2 weeks, 1 month, 3 months, 6 months, 12 months, and 24 months to cover important phases of bone development, ranging from early differentiation to bone maturation and aging. The experiments complied with the European Community Council directive (86/609/EEC) for the care and use of laboratory animals. Mice were euthanized via cervical dislocation, and both right and left hindlimbs were excised. Femurs and tibiae were cleaned of muscle tissue using a scalpel and gauze, then immediately placed in liquid nitrogen for processing. Bone was pulverized in a prechilled mortar using a pestle, and the powdered tissue was transferred to a microtube with NZYol reagent (1 mL) or TL buffer (0.4 mL) for RNA or DNA extraction, respectively.

### 4.6. RNA Extraction and cDNA Synthesis

Total RNA was extracted from cells and powdered bone using NZYol reagent, following the manufacturer’s instructions. RNA concentration and purity were determined with a NanoDrop One spectrophotometer (Thermo Fisher Scientific, Waltham, MA, USA), and integrity was assessed by electrophoresis on a 1.5% agarose gel stained with GreenSafe (NZYTech, Lisbon, Portugal). For *ZNF687* and osteogenic gene analysis, 1 μg of RNA was treated with RQ1 RNase-free DNase (Promega, Madison, WI, USA) for 30 min at 37 °C to remove gDNA, then reverse-transcribed for 50 min at 37 °C using Moloney murine leukemia virus (M-MLV) reverse transcriptase (Invitrogen). For miRNA expression analysis, 500 ng of RNA was polyadenylated and reverse-transcribed with the NCode miRNA First-Strand cDNA Synthesis Kit (Invitrogen).

### 4.7. Real-Time Quantitative Polymerase Chain Reaction (RT-qPCR)

RT-qPCR was conducted at least in duplicate in 20 μL reactions containing 10 μL of SensiFAST SYBR No-ROX mix (Meridian Bioscience, Cincinnati, OH, USA), 0.8 μL of primers (10 μM, Table S1), and 2 μL of 1:10 diluted reverse-transcribed RNA, using a CFX96 Connect Real-Time PCR machine (Bio-Rad, Hercules, CA, USA). Primers were designed using the PerlPrimer program with the following criteria: spanning an exon–exon junction; melting temperature of about 60 °C; amplicons between 100 and 200 bp; low self-complementary and 3′ complementary. The Primer-Blast Tool was used to ensure primer specificity, and primer efficiency was examined with a standard curve of Ct value vs. log (cDNA dilution). Amplification conditions included a polymerase activation step at 95 °C for 2 min, followed by 40 cycles of denaturation (5 s at 95 °C) and annealing/extension (20 s at 60 °C). The melting curve was performed at temperatures ranging from 65 °C to 95 °C and increments of 0.5 °C/s. Relative gene expression was calculated using the 2^−ΔΔCt^ method [66] and normalized to glyceraldehyde-3-phosphate dehydrogenase (*Gapdh*).

For miRNA expression analysis, qPCR was performed with 2 μL of reverse-transcribed RNA, 1 μL of primers (10 μM, Table S1), and Platinum SYBR Green qPCR SuperMix-UDG (Thermo Fisher Scientific), with conditions including uracil DNA glycosylase (UDG) incubation (2 min at 50 °C), polymerase activation (2 min at 95 °C), and 40 cycles of denaturation (5 s at 95 °C) and annealing/extension (30 s at 60 °C). miRNA expression was normalized to U6 small nuclear RNA. A negative control was prepared using water as the template.

### 4.8. Protein Extraction and Western Blot

To prepare protein samples, cells were lysed in buffer (50 mM Tris-HCl, pH 7.4; 150 mM NaCl; 1% Triton X-100) containing a complete protease inhibitor cocktail (Roche, Basel, Switzerland). After incubating on ice for 30 min, the lysates were centrifuged at 13,000× *g* for 15 min at 4 °C. The supernatants were then collected, and the protein was quantified using the Bio-Rad Protein Assay (Bio-Rad). The total protein was denatured in sample buffer (200 mM Tris-HCl pH 6.8; 9% 2-mercaptoetanol; 9% SDS; 40% glycerol; and 0.04% bromophenol blue) at 100 °C for 5 min. After that, 30 μg of protein was separated on a 4–12% acrylamide NuPAGE Bis-Tris gel (Invitrogen) and transferred to a PVDF membrane (Millipore, Burlington, MA, USA). The membrane was blocked in 5% non-fat milk (Blotting-Grade Blocker, Bio-Rad) in TBS-T for 1 h. According to the molecular weights ZNF687 and β-actin, the membrane was cut horizontally in two and each part was incubated overnight at 4 °C with the corresponding antibody: rabbit anti-ZNF687 (1:100, ProSci, Poway, CA, USA) and mouse anti-β-actin (1:10,000, Proteintech, Rosemont, IL, USA). The membranes were then washed with TBS-T and incubated for 1 h with anti-rabbit (1:10,000, Sigma-Aldrich) and anti-mouse (1:15,000, Sigma-Aldrich) IgG–peroxidade conjugate antibodies. The membranes were washed in 1× TBS-T and a chemiluminescent signal was detected with the Amersham ECL Western Blotting Detection Kit (Cytiva, Marlborough, MA, USA). Images were captured and visualized with ImageQuant LAS500 (Cytiva).

### 4.9. DNA Extraction and Bisulfite Conversion

Genomic DNA was extracted from cultured cells and powdered bone tissues using the E.Z.N.A. Tissue DNA Kit (ZYMO, Irvine, CA, USA) and quantified with a NanoDrop One spectrophotometer. The genomic DNA was then subjected to bisulfite modification using the EZ DNA methylation-gold kit (ZYMO), converting unmethylated cytosine to uracil while preserving methylated cytosine. Briefly, 500 ng of DNA was mixed and incubated with the CT conversion reagent at 98 °C for 10 min, followed by 2.5 h at 64 °C. This mixture was loaded onto a column, incubated with desulfonation buffer for 20 min, and following several washes, the bisulfite-converted DNA was eluted in DNase/RNase-free water.

### 4.10. PCR Amplification of Bisulfite-Converted DNA and Cloning into a TOPO Plasmid

Bisulfite-converted gDNA was PCR-amplified using primers targeting ten amplicons within the *Zfp687* gene containing CpG sites (Figure S4). Due to DNA fragmentation and the absence of cytosines following bisulfite conversion, primers were designed to avoid CpG dinucleotides, be 26–30 bases in length, and target amplicons of 149–3750 bp (primer sequences outlined in Table S1). PCR amplification was performed using the HotStarTaq Plus Master Mix kit (Qiagen, Hilden, Germany) in a C1000 Touch™ Thermal Cycler (Bio-Rad) under the following conditions: initial activation at 95 °C for 5 min, followed by 35 cycles of denaturation at 94 °C for 30 s, annealing at 50 °C for 30 s, and extension at 72 °C for 1 min, with a final extension at 72 °C for 10 min. PCR products were separated on a 1.2% agarose gel, and fragments were excised and purified using the GeneJET Gel Extraction Kit (Thermo Fisher Scientific).

Ligation of these fragments into the pCRII-TOPO vector (Invitrogen) occurred through the incubation of 4 μL of PCR fragments, 1 μL of salt solution, and 1 μL of the vector for 18 h at 4 °C. The ligations were then transformed into DH5α *E. coli*-competent cells by heat shock and spread on LB agar plates containing penicillin (50 μg/mL). Individual colonies were then cultured in liquid LB medium for 18 h at 37 °C. Plasmid DNA was purified using the NZYSpeedy Miniprep Kit (NZYtech) and screened for positive clones by endonuclease digestion with *Eco*RI. Ten positive clones from each sample were sequenced using the Sanger method at the Centre of Marine Sciences (CCMAR) with T7 and SP6 primers (Table S1) to determine the methylation levels of each CpG site.

### 4.11. Cloning of the Zfp687 3′UTR into the pMIR-Report Plasmid

The *Zfp687* 3′UTR fragment was amplified from MC3T3 DNA using specific primers (Table S1), with *Spe*I and *Hind*III restriction sites at the 5′ ends of the forward and reverse primers, respectively. The PCR reaction contained 50 ng of DNA, 1.5 μL of dNTPs (10 mM), 10 μL of 5× buffer, 1.5 μL of primers (10 μM), and 1 U of KAPA Hifi DNA polymerase (Roche) in a total volume of 50 μL. PCR conditions were as follows: initial denaturation at 95 °C for 3 min, followed by 35 cycles of denaturation at 98 °C for 20 s, annealing at 58 °C for 15 s, and extension at 72 °C for 40 s, with a final extension at 72 °C for 7 min. The PCR fragments were digested with *Spe*I and *Hind*III enzymes (5 U; Takara, Kusatsu, Japan) for 2 h at 37 °C, and purified from agarose gel. The ligation into the pMIR report vector (Promega) digested with *Spe*I and *Hind*III was performed at a 5:1 insert/vector ratio at 16 °C for 4 h using T4 DNA ligase (3 U, Promega). DH5α *E. coli*-competent cells were transformed with the ligation products, and plasmid DNA was extracted and purified using the ZymoPURE Plasmid Miniprep Kit (ZYMO). The construct (pMIR-ZNF687-3′UTR) was verified by sequencing.

### 4.12. PCR-Based Site-Directed Mutagenesis

The predicted binding site of miR142a-3p in the mouse *Zfp687* 3′UTR was mutated in the pMIR-ZNF687-3′UTR construct using the QuikChange Lightning Site-Directed Mutagenesis Kit (Agilent Technologies, Santa Clara, CA, USA). Mutagenic primers were designed using the QuikChange Primer Design Program (www.agilent.com/store/primerDesignProgram.jsp) (Table S1). The reaction included 50 ng of pMIR-ZNF687-3′UTR plasmid, 5 μL of 10× buffer, 1.25 μL of mutagenic primers (10 μM), 1 μL of dNTP mix, 1.5 μL of QuikSolution reagent, and 1 μL of QuikChange Lightning Enzyme in a total volume of 50 μL. Amplification conditions were: initial denaturation at 95 °C for 2 min; 18 cycles of denaturation at 95 °C for 20 s; annealing at 60 °C for 10 s; and extension at 68 °C for 4 min, followed by a final extension at 68 °C for 5 min. *Dpn*I was used to digest non-mutated DNA, and the mutated construct was transformed into XL10-Gold ultracompetent cells, amplified, and purified using the ZymoPURE Plasmid Miniprep Kit. Mutation presence was confirmed by sequencing with the M13 forward primer (Table S1).

### 4.13. Transient Transfection and Luciferase Reporter Assay

On the day before transfection, 5 × 10^4^ HEK-293 cells, 1 × 10^5^ MC3T3-E1 cells, and hFOB1.19 cells were seeded in 24-well plates to reach approximately 70% confluency. Transfections were performed with 1 μL of XtremeGENE HP DNA Transfection Reagent (Roche), 250 ng of pMIR-ZNF687-3′UTR constructs, and 5 ng of pRL-null (Promega) for Renilla luciferase normalization in culture medium without supplements. For co-transfections, 25 nM of the miR negative control or the miR-142-3p mimic (miRCURY LNA miRNA, BioNova Cientifica, Madrid, Spain) was added. The negative control miRNA is designed to lack targets in human, mouse, or rat genomes. Transfection mixtures were incubated for 15 min and added to the cells drop by drop. After 48 h of incubation at 37 °C, cells were washed with cold PBS and lysed in passive lysis buffer (Biotium, Fremont, CA, USA). The lysates were clarified by centrifugation at 14,000× *g* for 30 s.

Firefly and Renilla luciferase activities were measured using the Firefly & Renilla Luciferase Single Tube Assay Kit (Biotium). Bioluminescence was read using the Synergy 4 microplate reader (BioTek) and luciferase activity was expressed as the Firefly/Renilla ratio.

### 4.14. Statistical Analysis

Statistics were conducted using GraphPad Prism 8. The Kolmogorov–Smirnov test was applied to evaluate the data distribution. The statistics test applied for normally distributed data was a one-way analysis of variance (ANOVA) followed by Tukey’s post-test for three groups and student’s *t*-test for two groups. Data not normally distributed were analyzed with the Mann–Whitney U test for two groups and the Kruskal–Wallis test followed by the Dunn multiple comparisons test for three groups. Data with two independent variables were analyzed with a two-way ANOVA followed by Sidak’s multiple comparisons test. A *p*-value < 0.05 was considered to indicate statistical significance.

## Data Availability

Data will be made available on request.

## Supplementary Materials

The following supporting information can be downloaded at: https://www.mdpi.com/article/10.3390/ijms26052069/s1.

## References

[1] Florencio-Silva R., Sasso G., Sasso-Cerri E., Simões M.J., Cerri P.S. (2015). Biology of bone tissue: Structure, function, and factors that influence bone cells. Biomed. Res. Int..

[2] Chalmers G.L. (2011). Disorders of bone remodeling. Annu. Rev. Pathol..

[3] Rucci N. (2008). Molecular biology of bone remodelling. Clin. Cases Miner. Bone Metab..

[4] Robling A.G., Castillo A.B., Turner C.H. (2006). Biomechanical and molecular regulation of bone remodeling. Annu. Rev. Biomed. Eng..

[5] Katsimbri P. (2017). The biology of normal bone remodelling. Eur. J. Cancer Care.

[6] Blair H.C., Larrouture Q.C., Li Y., Lin H., Beer-Stoltz D., Liu L., Tuan R.S., Robinson L.J., Schlesinger P.H., Nelson D.J. (2017). Osteoblast differentiation and bone matrix formation in vivo and in vitro. Tissue Eng. Part B Rev..

[7] Wang L., You X., Zhang L., Zhang C., Zou W. (2022). Mechanical regulation of bone remodeling. Bone Res..

[8] Peel N. (2009). Bone remodelling and disorders of bone metabolism. Surgery.

[9] Gennari L., Rendina D., Falchetti A., Merlotti D. (2019). Paget’s disease of bone. Calcif. Tissue Int..

[10] Alonso N., Calero-Paniagua I., del Pino-Montes J. (2017). Clinical and genetic advances in Paget’s disease of bone: A review. Clin. Rev. Bone Miner. Metab..

[11] Silva I.A.L., Conceição N., Gagnon É., Brown J.P., Cancela M.L., Michou L. (2018). Molecular effect of an OPTN common variant associated to Paget’s disease of bone. PLoS ONE.

[12] Silva I.A.L., Varela D., Cancela M.L., Conceição N. (2022). Zebrafish optineurin: Genomic organization and transcription regulation. Genome.

[13] Divisato G., Formicola D., Esposito T., Merlotti D., Pazzaglia L., Del Fattore A., Siris E., Orcel P., Brown J.P., Nuti R. (2016). ZNF687 mutations in severe Paget disease of bone associated with giant cell tumor. Am. J. Hum. Genet..

[14] Scotto di Carlo F., Pazzaglia L., Mumm S., Benassi M.S., De Chiara A., Franchi A., Parafioriti A., Righi A., Esposito T., Whyte M.P. (2020). ZNF687 mutations in an extended cohort of neoplastic transformations in Paget’s disease of bone: Implications for clinical pathology. J. Bone Miner. Res..

[15] Russo S., Scotto di Carlo F., Maurizi A., Fortunato G., Teti A., Licastro D., Settembre C., Mello T., Gianfrancesco F. (2023). A mutation in the ZNF687 gene that is responsible for the severe form of Paget’s disease of bone causes severely altered bone remodeling and promotes hepatocellular carcinoma onset in a knock-in mouse model. Bone Res..

[16] Ganss B., Jheon A. (2004). Zinc Finger Transcription Factors in skeletal development. Crit. Rev. Oral Biol. Med..

[17] Varela D., Varela T., Conceição N., Cancela M.L. (2023). Regulation of human ZNF687, a gene associated with Paget’s disease of bone. Int. J. Biochem. Cell Biol..

[18] Park-Min K.H. (2017). Epigenetic Regulation of Bone cells. Connect. Tissue Res..

[19] Diboun I., Wani S., Ralston S.H., Albagha O.M.E. (2022). Epigenetic DNA methylation signatures associated with the severity of Paget’s disease of bone. Front. Cell Dev. Biol..

[20] Moss E.G. (2002). MicroRNAs: Hidden in the Genome Genes for tiny RNAs have been found to be plentiful. Curr. Biol..

[21] Bourne L.E., Wheeler-Jones C.P.D., Orriss I.R. (2021). Regulation of mineralisation in bone and vascular tissue: A comparative review. J. Endocrinol..

[22] Bernar A., Gebetsberger J.V., Bauer M., Streif W., Schirmer M. (2023). Optimization of the alizarin red S assay by enhancing mineralization of osteoblasts. Int. J. Mol. Sci..

[23] Luttrell L.M., Dar M.S., Gesty-Palmer D., El-Shewy H.M., Robinson K.M., Haycraft C.J., Barth J.L. (2019). Transcriptomic characterization of signaling pathways associated with osteoblastic differentiation of MC-3T3E1 cells. PLoS ONE.

[24] Collart-Dutilleul P.Y., Deville de Périère D., Cuisinier F.J., Cunin F., Gergely C. (2014). Porous silicon scaffolds for stem cells growth and osteodifferentiation. Porous Silicon for Biomedical Applications.

[25] Beck G.R., Zerler B., Moran E. (2001). Gene array analysis of osteoblast differentiation. Cell Growth Differ..

[26] Jiang Q., Nagano K., Moriishi T., Komori H., Sakane C., Matsuo Y., Zhang Z., Nishimura R., Ito K., Qin X. (2024). Roles of Sp7 in osteoblasts for the proliferation, differentiation, and osteocyte process formation. J. Orthop. Translat.

[27] Wang J.S., Kamath T., Mazur C.M., Mirzamohammadi F., Rotter D., Hojo H., Castro C.D., Tokavanich N., Patel R., Govea N. (2021). Control of osteocyte dendrite formation by Sp7 and its target gene osteocrin. Nat. Commun..

[28] Wrobel E., Leszczynska J., Brzoska E. (2016). The characteristics of human bone-derived cells (HBDCS) during osteogenesis in vitro. Cell Mol. Biol. Lett..

[29] De Barros T.L., Brito V.G.B., Do Amaral C.C.F., Chaves-Neto A.H., Campanelli A.P., Oliveira S.H.P. (2016). Osteogenic markers are reduced in bone-marrow mesenchymal cells and femoral bone of young spontaneously hypertensive rats. Life Sci..

[30] Maier T., Güell M., Serrano L. (2009). Correlation of mRNA and protein in complex biological samples. FEBS Lett..

[31] Vogel C., Marcotte E.M. (2012). Insights into the regulation of protein abundance from proteomic and transcriptomic analyses. Nat. Rev. Genet..

[32] Ezura Y., Sekiya I., Koga H., Muneta T., Noda M. (2009). Methylation status of CpG islands in the promoter regions of signature genes during chondrogenesis of human synovium-derived mesenchymal stem cells. Arthritis Rheum..

[33] Hu W., Ye Y., Zhang W., Wang J., Chen A., Guo F. (2013). MiR-142-3p promotes osteoblast differentiation by modulating Wnt signaling. Mol. Med. Rep..

[34] Chan W.C.W., Tan Z., To M.K.T., Chan D. (2021). Regulation and role of transcription factors in osteogenesis. Int. J. Mol. Sci..

[35] Ponzetti M., Rucci N. (2021). Osteoblast differentiation and signaling: Established concepts and emerging topics. Int. J. Mol. Sci..

[36] Choi Y.H., Jeong H.M., Jin Y.H., Li H., Yeo C.Y., Lee K.Y. (2011). Akt phosphorylates and regulates the osteogenic activity of Osterix. Biochem. Biophys. Res. Commun..

[37] Chen G., Deng C., Li Y.P. (2012). TGF-β and BMP signaling in osteoblast differentiation and bone formation. Int. J. Biol. Sci..

[38] Divisato G., Scotto di Carlo F., Petrillo N., Esposito T., Gianfrancesco F. (2018). ZNF687 mutations are frequently found in pagetic patients from South Italy: Implication in the pathogenesis of Paget’s disease of bone. Clin. Genet..

[39] Rutkovskiy A., Stensløkken K.-O., Vaage I.J. (2016). Osteoblast differentiation at a glance. Med. Sci. Monit. Basic. Res..

[40] Somerville J.M., Aspden R.M., Armour K.E., Armour K.J., Reid D.M. (2004). Growth of C57Bl/6 mice and the material and mechanical properties of cortical bone from the tibia. Calcif. Tissue Int..

[41] Silva M.J., Brodt M.D., Lynch M.A., Stephens A.L., Wood D.J., Civitelli R. (2012). Tibial loading increases osteogenic gene expression and cortical bone volume in mature and middle-aged mice. PLoS ONE.

[42] Cao J., Venton L., Sakata T., Halloran B.P. (2003). Expression of RANKL and OPG correlates with age-related bone loss in male C57BL/6 mice. J. Bone Miner. Res..

[43] Ferguson V.L., Ayers R.A., Bateman T.A., Simske S.J. (2003). Bone development and age-related bone loss in male C57BL/6J mice. Bone.

[44] Maupin K.A., Childress P., Brinker A., Khan F., Abeysekera I., Aguilar I.N., Olivos D.J., Adam G., Savaglio M.K., Ganesh V. (2019). Skeletal adaptations in young male mice after 4 weeks aboard the International Space Station. Microgravity.

[45] Halloran B.P., Ferguson V.L., Simske S.J., Burghardt A., Venton L.L., Majumdar S. (2002). Changes in bone structure and mass with advancing age in the male C57BL/6J mouse. J. Bone Miner. Res..

[46] Komori T. (2019). Regulation of proliferation, differentiation and functions of osteoblasts by runx2. Int. J. Mol. Sci..

[47] Arnsdorf E.J., Tummala P., Castillo A.B., Zhang F., Jacobs C.R. (2010). The epigenetic mechanism of mechanically induced osteogenic differentiation. J. Biomech..

[48] Villagra A., Gutirrez J., Paredes R., Sierra J., Puchi M., Imschenetzky M., Van Wijnen A., Lian J., Stein G., Stein J. (2002). Reduced CpG methylation is associated with transcriptional activation of the bone-specific rat osteocalcin gene in osteoblasts. J. Cell Biochem..

[49] Yu F., Shen H., Deng H.W. (2017). Systemic analysis of osteoblast-specific DNA methylation marks reveals novel epigenetic basis of osteoblast differentiation. Bone Rep..

[50] Shi K., Lu J., Zhao Y., Wang L., Li J., Qi B., Li H., Ma C. (2013). MicroRNA-214 suppresses osteogenic differentiation of C2C12 myoblast cells by targeting Osterix. Bone.

[51] Han X., Fan Z. (2021). MicroRNAs regulation in osteogenic differentiation of mesenchymal stem cells. Front. Dent. Med..

[52] Xu Y., Li D., Zhu Z., Li L., Jin Y., Ma C., Zhang W. (2020). MiR-27a-3p negatively regulates osteogenic differentiation of MC3T3-E1 preosteoblasts by targeting osterix. Mol. Med. Rep..

[53] Huang W., Paul D., Calin G.A., Bayraktar R. (2023). MiR-142: A master regulator in hematological malignancies and therapeutic opportunities. Cells.

[54] Kelch S., Balmayor E.R., Seeliger C., Vester H., Kirschke J.S., Van Griensven M. (2017). MiRNAs in bone tissue correlate to bone mineral density and circulating miRNAs are gender independent in osteoporotic patients. Sci. Rep..

[55] Letarouilly J.G., Broux O., Clabaut A. (2019). New insights into the epigenetics of osteoporosis. Genomics.

[56] Fröhlich L.F. (2019). MicroRNAs at the interface between osteogenesis and angiogenesis as targets for bone regeneration. Cells.

[57] Condrat C.E., Thompson D.C., Barbu M.G., Bugnar O.L., Boboc A., Cretoiu D., Suciu N., Cretoiu S.M., Voinea S.C. (2020). MiRNAs as biomarkers in disease: Latest findings regarding their role in diagnosis and prognosis. Cells.

[58] Huang J., Chen D. (2014). MiRNAs in circulation: Mirroring bone conditions?. J. Bone Miner. Res..

[59] Faraldi M., Gomarasca M., Banfi G., Lombardi G. (2018). Free circulating miRNAs measurement in clinical settings: The still unsolved issue of the normalization. Adv. Clin. Chem..

[60] Evans C.H. (2010). Gene therapy for bone healing. Expert. Rev. Mol. Med..

[61] Oh W.T., Yang Y.S., Xie J., Ma H., Kim J.M., Park K.H., Oh D.S., Park-Min K.H., Greenblatt M.B., Gao G. (2023). WNT-modulating gene silencers as a gene therapy for osteoporosis, bone fracture, and critical-sized bone defects. Mol. Ther..

[62] Yang Y.S., Xie J., Wang D., Kim J.M., Tai P.W.L., Gravallese E., Gao G., Shim J.H. (2019). Bone-targeting AAV-mediated silencing of Schnurri-3 prevents bone loss in osteoporosis. Nat. Commun..

[63] Lewis B.P., Burge C.B., Bartel D.P. (2005). Conserved seed pairing, often flanked by adenosines, indicates that thousands of human genes are microRNA targets. Cell.

[64] Chen Y., Wang X. (2020). MiRDB: An online database for prediction of functional microRNA targets. Nucleic Acids Res..

[65] Rennie W., Liu C., Carmack C.S., Wolenc A., Kanoria S., Lu J., Long D., Ding Y. (2014). STarMir: A web server for prediction of microRNA binding sites. Nucleic Acids Res..

[66] Schmittgen T.D., Livak K.J. (2008). Analyzing real-time PCR data by the comparative CT method. Nat. Protoc..

