# Supplementary material for: Epigenetic Regulation of ZNF687 by miR-142a-3p and DNA Methylation During Osteoblast Differentiation and Mice Bone Development and Aging
Source: Int J Mol Sci. 2025 Feb 27;26(5):2069. doi: 10.3390/ijms26052069 (PMC11899743; doi:10.3390/ijms26052069)
Supplement: Supplementary file 1 [file ijms-26-02069-s001.zip › Supplementary Figure S1.pdf]

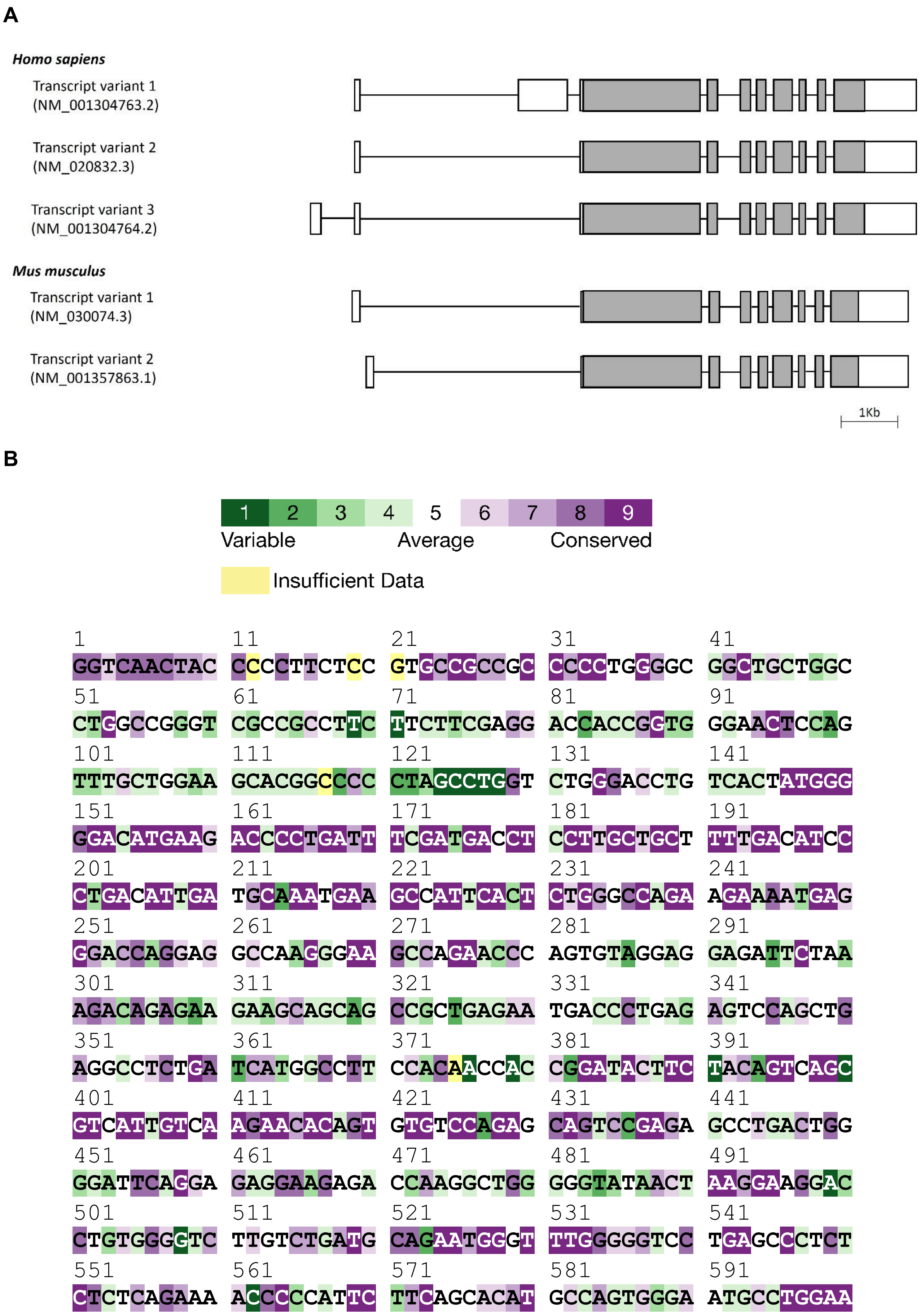
A
Homo sapiens
Transcript variant 1
(NM_001304763.2)
Transcript variant 2
(NM_020832.3)
Transcript variant 3
(NM_001304764.2)
Mus musculus
Transcript variant 1
(NM_030074.3)
Transcript variant 2
(NM_001357863.1)
1Kb
B
1 2 3 4 5 6 7 8 9
Variable
Average
Conserved
Insufficient Data
1 GGTCAACTAC 11 CCCCTTCTCC 21 GTGCCGCCGC 31 CCCCTGGGGC 41 GGCTGCTGGC
51 CTGGCCGGGT 61 CGCCGCCTTC 71 TTCTTCGAGG 81 ACCACCGGTG 91 GGAACTCCAG
101 TTTGCTGGAA 111 GCACGGCCCC 121 CTAGCCTGGT 131 CTGGGACCTG 141 TCACTATGGG
151 GGACATGAAG 161 ACCCCTGATT 171 TCGATGACCT 181 CCTTGCTGCT 191 TTTGACATCC
201 CTGACATTGA 211 TGCAAATGAA 221 GCCATTCACT 231 CTGGGCCAGA 241 AGAAAATGAG
251 GGACCAGGAG 261 GCCAAGGGAA 271 GCCAGAACCC 281 AGTGTAGGAG 291 GAGATTCTAA
301 AGACAGAGAA 311 GAAGCAGCAG 321 CCGCTGAGAA 331 TGACCCTGAG 341 AGTCCAGCTG
351 AGGCCTCTGA 361 TCATGGCCTT 371 CCACAACCAC 381 CGGATACTTC 391 TACAGTCAGC
401 GTCATTGTCA 411 AGAACACAGT 421 GTGTCCAGAG 431 CAGTCCGAGA 441 GCCTGACTGG
451 GGATTCAGGA 461 GAGGAAGAGA 471 CCAAGGCTGG 481 GGGTATAACT 491 AAGGAAGGAC
501 CTGTGGGGTC 511 TTGTCTGATG 521 CAGAATGGGT 531 TTGGGGGTCC 541 TGAGCCCTCT
551 CTCTCAGAAA 561 ACCCCCATTC 571 TTCAGCACAT 581 GCCAGTGGGA 591 ATGCCTGGAA

601	611	621	631	641
AGACAAAGCT	GTGGAAGGCA	AAACATGCTT	GGACCTCTTT	GCTCATTTTG
651	661	671	681	691
GGTCTGAACC	AGGAGACCAC	CCAGATCCCC	TACCTCCAGA	ACCTTCCCAA
701	711	721	731	741
CCTCGGGGTG	GTGACATGGC	CCCACCTCCT	TTCTCCACTC	CTTTTGAACT
751	761	771	781	791
GGCCCCAGAA	AATGGCTCAA	CCCTGCTTCC	TCCTGCTTCA	CTCTTGCCTC
801	811	821	831	841
AAGGGGCCTT	GAAACAGGAA	AGCTGCAGCC	CTCACCATTC	CCAGGGCCTA
851	861	871	881	891
ACCCAGAGAG	GCCCAGGCTC	CAGCCCCGAG	ACAGCAGGCA	TCCCTGCCAG
901	911	921	931	941
TGTCTCTCCC	CCGCAGGTGG	CTGGGGTGTC	CTTCAAGCAG	TCTCCAGGAC
951	961	971	981	991
ACCAGAGTCC	TCCTGCTTCC	CCTGTCAAGG	CACCCAGCTG	TAAACCCCTG
1001	1011	1021	1031	1041
AAGGAAGAAG	ATGAGGGAAC	AGTGGACAAG	TCTCCCCCAA	GAAGTCCCCA
1051	1061	1071	1081	1091
GAGTCCCTCT	AGTGGAGCTG	AGGCTGCAGA	TGAGGACAGC	AATGATTCCC
1101	1111	1121	1131	1141
CTACCTCCTC	CAGCTCTTCT	AGGCCCCTCA	AGGTGCGGAT	CAAGACTATT
1151	1161	1171	1181	1191
AAAACATCCT	GTGGGAATAT	CACAAGAACT	GTAACCCGGG	TCCCCTCAGA
1201	1211	1221	1231	1241
ACCCGATCCT	CCTGCCCCTT	TGGCCGAGGG	GGCTTTCCTG	GCCGAGACTA
1251	1261	1271	1281	1291
GCTTCCTGAA	ACTGTCCCCT	GTAACTCCAA	CCCCCGAAGG	TCCAAAGGTG
1301	1311	1321	1331	1341
GTGAGTGTCC	AATTGGGTGA	TGGCACGAGG	CTGAAAGGTA	CAGTGCTGCC
1351	1361	1371	1381	1391
TGTGGCCACC	ATCCAGAATG	CCAGCACTGC	CATGCTCATG	GCAGCCAGTG
1401	1411	1421	1431	1441
TAGCCCGCAA	AGCTGTGGTT	CTACCAGGGG	GGAATGCCAC	CAGCCCCAAA
1451	1461	1471	1481	1491
ACTATGACTA	AGAGTGTGTT	AGGTCTGGTG	CCCCAAACTC	TGCCCAAGGC
1501	1511	1521	1531	1541
TGAGGTACGG	ACAGGGTTTA	GCCTTGGGGG	GCAGAAGGTA	AATGGTGCCT
1551	1561	1571	1581	1591
CAGTGGTGAT	GGTACAGCCT	TCCAAGTCTG	CTACTGGGCC	AGGCACAGCA
1601	1611	1621	1631	1641
GGTGGCTCGG	TGATCTCCCG	AACCCAGTCC	AGTCTGGTAG	AGGCCTTCAA
1651	1661	1671	1681	1691
CAAGATCCTC	AACAGCAAGA	ACCTGCTGCC	TGCTTATAGA	CCAAACCTGA
1701	1711	1721	1731	1741
GTCCGCCAGC	TGAGGCTGGT	CTGGCCCTGC	CTCCAACAGG	CTACCGCTGC

1751	1761	1771	1781	1791
CTTGAGTGTG	GAGATGCCTT	CTCTCTGGAG	AAGAGCCTGG	CACGGCACTA
1801	1811	1821	1831	1841
TGATCGAAGG	AGCATGCGTA	TAGAGGTCAC	CTGTAACCAC	TGTGCCCGTC
1851	1861	1871	1881	1891
GCCTGGTTTT	CTTCAACAAG	TGTAGCCTGC	TTCTGCATGC	CCGTGAGCAC
1901	1911	1921	1931	1941
AAGGACAAGG	GGCTTGTCAT	GCAGTGCTCA	CATTTGGTCA	TGAGGCCTGT
1951	1961	1971	1981	1991
AGCCCTTGAC	CAGATGGTGG	GGCAGCCAGA	CATCACACCC	TTGCTGCCTG
2001	2011	2021	2031	2041
TGGCTGTCCC	ACCTGTTCCT	GGACCTTTGG	CCTTGCCTGT	TTTGGGCAAG
2051	2061	2071	2081	2091
GGGGAGGGGG	CTGTCACTTC	CTCTACCATC	ACTACAGTTG	CCACTGAAGC
2101	2111	2121	2131	2141
TCCTGTGCTG	CCACTCCCAA	CAGAGCCCCC	TGCTCCCCCT	ACTGCCTCTG
2151	2161	2171	2181	2191
TTTACACGTG	CTTTCGCTGT	CTGGAGTGCA	AAGAGCAGTG	CCGGGACAAG
2201	2211	2221	2231	2241
GCTGGCATGG	CAGCCCATTT	CCAGCAGCTG	GGCCCTCCTG	CACTCGGGTC
2251	2261	2271	2281	2291
TACCAGCAAT	GTGTGTCCGT	CCTGCCCCAT	GATGCTCCCC	AATCGCTGCA
2301	2311	2321	2331	2341
GCTTCAGTGC	CCACCAGCGC	ACACATAAGA	ATCGAGCCCC	CCATGTGTGT
2351	2361	2371	2381	2391
CCCGAGTGTG	GGGGTAACTT	CCTACAAGCT	AATTTTCAGA	CCCATCTTCG
2401	2411	2421	2431	2441
AGAGGCCTGT	CTGCATTTCT	CTCGCCGTGT	AGGATACAGG	TGCCCTAGCT
2451	2461	2471	2481	2491
GTGCAGTGGT	GTTTGGGGGT	GTGAACTCCA	TCAAGTCCCA	CATCCAGGCA
2501	2511	2521	2531	2541
TCACACTGCG	AAGTTTTCCA	CAAGTGCCCC	ATCTGCCCCA	TGGCCTTCAA
2551	2561	2571	2581	2591
GTCTGCACCC	AGCGCCCATG	CCCACCTCTA	CTCCCAGCAT	CCAAGCTTCC
2601	2611	2621	2631	2641
TCACCCAGCA	AGCCAAGCTG	ATCTATAAGT	GTGCCATGTG	TGATACGGTC
2651	2661	2671	2681	2691
TTCACTCACA	AACCCCTCCT	CTCCTCACAC	TTTGACCAGC	ACTTACTGCC
2701	2711	2721	2731	2741
CCAGCGTGTC	AGTGTCTTTA	AGTGCCCGTC	TTGTCCTCTG	CTTTTTGCCC
2751	2761	2771	2781	2791
AAAAAAGAAC	CATGCTGGAG	CATCTCAAGA	ACACTCACCA	GTCTGGACGT
2801	2811	2821	2831	2841
GTGGGGGAAG	AGGCAGTTGG	GAAAGGGGCT	GGAGGTGCCC	TTTTGACCCC
2851	2861	2871	2881	2891
CAAGACTGAG	CCTGAGGAGC	TGGCTGTGTC	TCAGGCAGAG	GCAGCCCCTG

2901	2911	2921	2931	2941
CTACTGAGGA	GTCTTCCTCA	TCTTCTGAAG	AGGAGCTGCC	TAGCTCCCCT
2951	2961	2971	2981	2991
GAGCCACCCC	GACCAACCAA	AAGAGCCCGA	CGAGGAGAAC	TGGGAAACAA
3001	3011	3021	3031	3041
AGGCATCAAA	GGTGGGGGTG	GGGGGCCTGG	GGGCTGGACT	TGTGGCCTTT
3051	3061	3071	3081	3091
GTCACTCCTG	GTGTCCTGAG	CGTGACGAGT	ATGTGACTCA	CATGAAGAAG
3101	3111	3121	3131	3141
GAACATGGTA	AGTCAGTGAA	AAAGTTCCCC	TGTCGCCTGT	GTGAGCGCTC
3151	3161	3171	3181	3191
CTTTTGCTCT	GCCCCAAGCC	TGAGGCGCCA	TGTCAGGGTC	AACCATGAGG
3201	3211	3221	3231	3241
GAATCAAGCG	AGTTTACCCA	TGCAGGTATT	GTACAGAGGG	AAAGCGCACC
3251	3261	3271	3281	3291
TTCAGTAGTC	GCCTGATCCT	GGAGAAGCAT	GTTCAGGTCC	GGCACGGCTT
3301	3311	3321	3331	3341
GCCTCTTGGG	ACCCAGTCTT	CTGGCCGAGG	AGGCTCCCTG	GCTCGAGGCT
3351	3361	3371	3381	3391
CTGGTGGCAG	AGCCCAGGGG	CCAGGACGGA	AACGCCGCCA	GTCTTCTGAC
3401	3411	3421	3431	3441
TCATGCAGTG	AGGAGCCTGA	CAGTACAACA	CCACCAGCCA	AGTCCCTGAG
3451	3461	3471	3481	3491
GGGTGGCCCT	GGGTCAGGAG	GCCACGGTCC	TCTGCGCTAT	AGAAGCAGTG
3501	3511	3521	3531	3541
GCTCAGCAGA	ACAGAGCCTT	GTGGGGTTGA	GGGTGGATGG	TGGCACTCAG
3551	3561	3571	3581	3591
CAGTGCCTTG	ACTGTGGCTT	GTGCTTTGCT	TCCCCTGGTT	CCCTGAGCCG
3601	3611	3621	3631	3641
CCACCGGTTC	ATTAGCCACA	AGAAGAGACG	GGCCGGGGGT	AAGGCCAGTG
3651	3661	3671	3681	3691
TCCTGGGGCT	GGGGGATGGG	GAAGAAGCAG	CTCCTCCTTT	ACGCTCTGAC
3701	3711	3721	3731	3741
CCAGAGGGTG	GAGACTCACC	TTTGCCTGCT	CCTGGAGACC	CTCTGACTTG
3751	3761	3771	3781	3791
TAAGGTCTGT	GGCAAGAGCT	GTGACAGCCC	TCTAAACCTC	AAGACCCATT
3801	3811	3821	3831	3841
TCCGCACGCA	TGGCATGGCA	TTCATCAGGG	CCCGGCAGGG	AGGCAGTGGG
3851	3861	3871	3881	3891
GACAACTAGG	TCCCCAGCCT	CAGACTGACC	AGGCCTCTTC	CCTGGGACTT
3901	3911	3921	3931	3941
GGGTTTCACT	GCTGCTGTCA	CCTCCCTGGG	CTGGGAGTTT	AACATTTACT
3951	3961	3971	3981	3991
TCTTTCTCTA	TCCCTGAGGG	ACAGGCTTTT	GAGGATTCTA	GTGACTTGCA
4001	4011	4021	4031	4041
CCCCCTTCCC	TCTAGGGTTT	GGCCCCTAGG	TTCTACTTAA	GTGGGCCTTC

4051	4061	4071	4081	4091
TTTTTCCTTT	CTGAATCCAA	CACTAATGCT	CCTGCTGCAG	ACTCCCAGTA
4101	4111	4121	4131	4141
ACTGTGGGGG	GAGGGGGGAG	GGGACCTTGG	CAAGCCTGCT	AGACAGGCAT
4151	4161	4171	4181	4191
AGGGATGGAC	TGAAGGATGT	ACAGGGTCTA	GCTTCTACCC	CTCTACTAGA
4201	4211	4221	4231	4241
GTTCTTCAGG	CACAGACCAG	TATTTCTTCT	TCTGGGCTGG	GTTCTGTCCT
4251	4261	4271	4281	4291
AGTATCCCCT	CCTCCAACAC	TCATTGGCAC	TAACACCCCC	ACCCCAATCC
4301	4311	4321	4331	4341
CTGCAGTGCC	TTCCATTTGT	CTTTTCCCCC	AGAAATTTAG	GGGTGGGGGG
4351	4361	4371	4381	4391
TTGGTGGGGA	TGAGTCCTGT	CAAGTAGGTT	CCAGGGAGAG	GAGAGGACAG
4401	4411	4421	4431	4441
GCTCTCAGGA	ATCCTTTATT	CTTGTAGTAG	TAGTAATACT	AACAGTGGGA
4451	4461	4471	4481	4491
GAAACAGGAG	GGAGAAAACC	AGACCATTAA	AACTGCTTGT	GGTTTAATCC
4501	4511	4521	4531	4541
CCATTCAGGC	TTTTCTTTTT	ATGTGAGTTG	GATAATGTGT	ACTCATAGGG
4551	4561	4571	4581	4591
AGGGGCGAGT	CAGAACTGGG	GAAGCCCTTC	TCAGGGAAGA	GGCAGTATTG
4601	4611	4621	4631	
ACGAAGACAG	ATGGAAAAAG	CAATCTTCCT	TC	

Supplementary Figure S1. *Zfp687* gene conservation. A) Schematic representation of the human *ZNF687* and mouse *Zfp687* transcript variants. Human *ZNF678* and mouse *Zfp687* have three and two transcript variants, respectively. Grey boxes indicate the coding region and the white boxes indicate the 5' and 3' untranslated regions. Exons and introns of human transcript are in scale. Exons of mouse transcripts are in scale while introns are not. **B)** Evolutionary conservation scores for each nucleotide position of mouse *Zfp687* (NM_001357863.1) across 123 species, analyzed with ConSurf tool.
