# Supplementary material for: Epigenetic Regulation of ZNF687 by miR-142a-3p and DNA Methylation During Osteoblast Differentiation and Mice Bone Development and Aging
Source: Int J Mol Sci. 2025 Feb 27;26(5):2069. doi: 10.3390/ijms26052069 (PMC11899743; doi:10.3390/ijms26052069)
Supplement: Supplementary file 1 [file ijms-26-02069-s001.zip › Supplementary Figure S2.pdf]

```
Mus musculus Zfp687 (NC_000069.7)  CTTGCTTGGACTGGGTGAAGCAC--TAGG---TTCCACTCCGAACACTGCAAAAGTGAAA 94923937
Homo sapiens ZNF687 (NG_051575.1)  CTCACTTGTAGTCCCAGCTACTCAGGAGGCTGAGGCGGGAGGATCACTGGAGCCTGGGAGG 4532
                                   **  **** * *    *   * *   ***      *    ** ***** *    * **
```

```
Mmus   ATAAAACAGAAATTTAAAAACCCTTGTCATTTGAAACGGCTGCGGT------CGCGGTGGGCGGCGGCG-------------------------------GCGCGGGGCCAGGGTAGAGA 94923854
Hsa    TTGAGGCTGCAGTC----AGCCGTGATCGCGCCACTGTGCTCCCGCCTGGGCAGCAGAGTGAGGCTCTGCCTCAAAAAAATTAATTAAAAAATAAAAACACGCCGTTAACGAGCGGCTGC 4648
        * *  * * * *     * ** *  **     *    *** * *        ** * * * ***   *                                  **    *  *     *

Mmus   GCGCGCCAGGTGGCACACTGGGCCTGCAGATGGATCCAGCAAAAACCTGAGCCAGCGCAGGTGGGTGAGTGTCAAGGCAATTCGTCTCCCTGGCGACTCCGCCCCTTTTCCCACCTCCAA 94923734
Hsa    GTGCGCGAGGTGGCACACTGGGCTTGCATACGAATTCTG-----------CCAGTGAGTGCTATTAGAGCATCAAGGAGATTCATTTTCCGAGCGACTCCTCCCTTTCCCCCGCCTCCAG 4757
       * **** **************** **** * * ** * *            *       * *    ***  ******  **** * * **  ******** *** **  *** ******

Mmus   GGGGCGGGGCAGCAAAGCGCGGGAAAGCTCAACGGGAACAGTAATGCCAGCTCCCGTCAGGGGCGGAGAAAAGGGAGCTCTCTTAAATGGGAAATAGATCTGGGCTCCCTCCTTCTCTCG 94923614
Hsa    GGGGCGTGGCAACAAAGCGCGGGAAAACTCGACCTTGACCCCACCCCACTCCATGCTGGGGGCGG-GAAAAGGGGAGCCCTTAAATGGGAAATGGAGCTGGCGGTTCCCCCCCCAGCC 4876
       ****** **** ************** *** **    **   *   ***   *  *   *******   *** ** *  * ************** ** ****     * **  *   *

Mmus   C----GTAAGAAGCCTATCAGCATACTACCTTAAGCTCTCCTTGGTTGTGGCCCAGGCCACGCCAGCACTTCCCAACGTCACCTCCCCCACTTCCGGTCGCAGCGTGTCCCAGGTCTCAG 94923498
Hsa    CATACTTAGGACGCCTATCAG-TTGCTCCTTTAATGCCTCTTTGGAAATG-GCCCGGCCACGCCAGCACT--CCTACGTCACCTCCCCCGCTTCCGGTCCGCGAGCCCTTCC-------C 4985
       *     ** ** *********  * ** * ****   *** ****   **  ** ***************  ** ************** *********   * *     *

Mmus   GTCTCGGTGGTTCCGGTTTCTTTTACACTCGGAATCCCAAAGTCTG-TCCAGCTCTCCCGCAGAGGGAGGGGCCGTGCGTGGTGCGTACGCCCAGAGGCAGGCGGACGCCAGAACCGCAT 94923379
Hsa    AACCTTTAGGTCCCGATTCCTTCCATGCTCCAAATCCCGTGCCCCGTCCACGCCCTCCCGCAGAGGGAGGAGCGACGGGTTACGCTGTCGCCCAGGAGCTGAACCGCGCGAGGACCCCAT 5105
         *     *** *** ** ***  *  ***  ******     * *  *  ** **************** **   * **   **   *******  ** *     *** ** *** ***

Mmus   CCATCCGGATTATAAAGCAGTTTAGACTGCGA-GGAACCCCAGGCAATGGTCACCCCGATGACGTAATGTTTGGGGGTGGCACCTCATTTTGTGACCCCCAGCACCGCATCCGTTCGAGC 94923260
Hsa    CCATCAGATT-ATATGGCGATTTAGACGGTGGGAAGACCGCAAGGAAATGGTCAGCGGATGACGTAATGTTTGGGGTGGCGTC-CCATTCTGTAACTTC-TGTACGGCATCAGTGACA-- 5220
       ***** *  * ***  **  ******* * *     *** ** * **  *     * ******************* *   *  **** *** **  *  * ** ***** **   *

Mmus   GCGGCTTAGCCTGAGGAAACGGCACGAACACTTAAAACACCCAGGTTCCACACGGAGCCGACGAGGTTCCCCTAGACG----CGCCGCCATACAGCTAGAGTTCCTAAGTTTGTAGATGT 94923144
Hsa    CGGGTTTAGCTTGAA-ACAGCGCAAAAGGACCTAC-ACTTCTGTGCTCCACTCCGACTCAATGCGGTTCTGCTCTCAGTGCCCACCACACAGAAGCTGGGTTCCTTACATTCGCGAAAGT 5338
         ** ***** ***  * *  ***  *  ** **  **  *   * ***** * **  * * * *****  **    *    * ** *     **** *  * * **  ** *   * **
```

```
Mmus  GAGGCACAGTGTCCAGCTTCGAGCGACCAATGGAGAGAGGATGGCTACGCTTCTGAGCTAGCTAGTCACCCCTCGCAACCCAAAGCGAAGACTAAGGCTGGCTTCTTGCTAAGCGACAGC 94923024
Hsa   GAACTGCAGCTTCAAGCTTCGAGCGGCCAGTAGGGAGCGAGGG--GCGGAGACGCACCTGGGTGCTCCCCCTTCCAGTAGGCAAGCTGGGAAGCAGGCTGGCTTCTTTCTAAGCGACAGT 5456
      **    ***  **  ***********  *** * * *** *    *      *    *  * ** * *   ** *** **        ****    **    ************* ***********

Mmus  GGCGGCGGCCAATGGCTTTCGGGCTGCTCAGACAGGGCAGGTGGACTCCGGAGCAGCCAATGGGGAGAACCTTCTCCACAGGGCCCAGGCCCTTTGTCCAATAGTGATGAGAAGACTATG 94922904
Hsa   GGCGGAGGCCAATCGTTTACGAGACTCGTAGATGGGGCAGACGGACCCCGGCGCAGCCAATGGGGAGAGGTATCTTCAGAGGGCCTCGGCCCCTTGCCCAATGAGCATAAGAGGACTGTG 5576
      ***** ******* * ** ** *    *   ***    ******    **** **** ****************     *** ** ******   ***** *** *****     ** *** **** **

Mmus  AGGGGCCAGTTCCGAGCGGGG-TATGGAGGGCCAATCCCATACGGGACTGGGTGGGGCTCACAGTTGAATGACAGGTCCATTAGCCAATGGCGAGTATGAAAGCGGGCAGCCCGGACTAG 94922785
Hsa   CGGGGCTAGTTCCGAGCGGCAGTATTTAGGGCCAACCCAGTGCGGGGCTGGAAGGGGCACAGGGCTGAGCGACGGGGGCAAGGGCCAATGGCGAGTGCGAAAACGGGCAGCCCAGACCTG 5696
       ***** ***********    ***   ******** **    * **** ****   ***** **    * ***   *** **   **    *************  **** ********** ***  *

                       +1
Mmus  GAGCACGGAGGGAGCTGAGGGAGAGGGGAGGCGAAGCGGAGAGAAGCAGGAAGTAGCGGCGGCCGCGGGGAGGGCGGCGGCGGCGGTAGCGGCGGTGGCGGCTGGAACCGGGAGGAGGC 94922665
Hsa   GAGCGCGGAGGGAGGAGAGGGAGTGGGGAGGCGAACCGGAGAGAAGCAGGAAGTAGCGGCGGCCGCGGGGAGGGCGGCGGTGGCTGCAG---------CGGCTGGAGCGGGGTAGAGAC 5807
      **** *********  ******* ************ ******************************************** *** * **         ******** * ***  *** *

Mmus  CGTAGGGTCTCAGCCCGCACCAGGATCGGAACAAGTAAGCACGCTCGCGGCAAATACCCGCCCCTGGCTCCGCCCCCCGG--GGGGGCGGGTAAATACCCCTGCCCCTCCCCCACTTGGT 94922547
Hsa   CGCCGGGTCTCGGCCCGCACCAGGAGCGGAACAAGTAAGCGTGCCTGGGGTAAATACCCGCCCTTGGCTCCGCCCCCTTGGGGGGGCGGGTAAATACCCCCGCCCCTCCCCCACTTGGT 5927
      **  ******* ************* **************   **   * ** *********** ************  *   ****************** *****************

Mmus  CAACTACCCCCTTCTCCGTGCCGCCGCCCCCTGGGGCGGCTGCTGGCCTGGCCGGGTCGCCCGCCTTCTTCTTCGAGGACCACCGGTGGGAACTCCAGTTTGCTGGAAGCACGGCCCCCTA 94922427
Hsa   CAACTACCCCCTTCTCCGCGCCGCCCCCTGG---GGCGGCTTCTAGGAAGGCCCAGACACCGCCTCCATCCATTGGGGCGGATGGGCGAAACTCCTGGCCTGTTGTAAGCACAGTCCGTA 6044
      **************** ****** **      ******* ** *    ****  * * ****** * **     ** *     **  * ****** *     * * *        ** **

Mmus  GCCTGGTAACGTGGGAGGGGGTTCCTGGACCCCTAAGTCCTGGCGCTCTTAGACTGAGAGGCAGTCAGAGTGCGGCCGGGGGACTCGAGGCTGCGGGATGTCGCGACCTAAAATGGTGTC 94922307
Hsa   GCCGGGTAAACCCCCA-GGAGGGTCGAGAACCCCAGGTCCTGGCGCCCCCGGCTCGAGAGGCACCCGAAGTGCGGCCCGGAGATTGAAGGCTGCGGGATGTCGCGACCTAAAATGGTGTC 6163
      *** *****       * ** *   *   ** *** * ********** *   *    ********   *   ********* ** ** *   *********************************

Mmus  CGTGCCGGGCGGTGGGGGCGGGGGAATTGGGCCCAGCAGGAGAGGAGCCTGCATCCCGACCGGGTGCTGGTCAGCGTCCGGACCCAGGCAGGACTCAGGCTAGCGCCTCGATCCTGGCGA 94922187
Hsa   CGTTCCCGGGGATGGGGGCGGGGGACCTGGGCCAGGCCGGAGCAGAACCTGCACCCCGACTGGGTGCGGGTCAGCGTCCGGCCCGGGCAAGACTCAGGCTTGCGCCCCCGGCCCCCTGC 6283
      *** ** ** * *************   ******   ** ****   ** ****** ****** ****** ************* *** **** ********** ***** *    **    *
```

```
Mmus    GCTCCTTGCAGTCACTCGGGT--CTGGGGAGAGCCTCTGGGGGTCAGAGACAGGGCAAGCTGCCCGGCGCGCAGAGATGAAGGGGACCAGGTTCGAGTCTGGAC-TGACCTGGCTGCAGA 94922070
Hsa     GCACCCCCCGATCAGTCTCCAGTCTCTAGCGAGCCTCCGGGAGTGCGCGGCGGGAGGAGGCTCCGCTGCGCCCGGAGGGGAGGGGACGAGGTCCGAGTTGGGACGGACCCCAGCTAGAGA 6403
        ** **   *  *** **   **   * ******* *** **  * * * **   **   **       * *    * ******* **** *****  ****    **  ***  ***

Mmus    GCTTAGAATCACGCAGGGACCGCCCCTCTTGTGTCTCCACTCCCCTCGCGCCACTCCCCACTGCCGGGGAGTCCTGGCCCCCAATGTGCAGCGCCGCAGATCCTAAG-GGCCTGGCCTC 94921951
Hsa     GCTGAGAGTCGCGCAGAGACCGTCCCTCTCTCCTCCCCTCTTTCCA--GCTCCCCACCCTATCCTGTGTAGTCCTGGGTCCCCAGGTGCTGCGCAGCAGCTCCCAAAGGGCCTGGCCTG 6521
        *** *** ** ***** ***** *******    ** ** **  **      * *  ***  * * * * ********  *** * **** **** **** *** **  **********

Mmus    ACCGAGGCCCTCCCTT-GAGTTCTTAGCCGGTGAAAGTGAAAGAAGCACCACTCGCCTCAGTTTACCCTTTAGTTTTTA-AATGTACTTCTTGTCCCCTACCCGAAACCCCGGACTTACT 94921833
Hsa     GCTGAGGCCCGGCCTTCGCTTCGCACGCCGGTGCGAGTGAGAGAAGGGCCGCGCGCCTCAGTTTCCCTTGTAGTTTCTCATGTGTACACCCCGCCCCCTCCTCGAAACCCAGGACTTGCT 6641
         * *******  **** *  *     *******  ***** *****  ** * *********** ** * ****** *    *****  *  * ***** * ******** ****** **

Mmus    TTCCGCGCCAGTCCCTGGCAGATGGCAGGGACTTAAATGCCGCCCGCTGCCCAGCTTGGCCGCAACCTAATCGCCCCTGGCCGTGCTCTGGCCCCTTCCTTTTGCTCTGCGCTGTAGGCC 94921713
Hsa     CCCCGCGCCAGCCCTCGGCAGATGGCAGGGACTTAATTC-CGTCTGCTACCCAGCTTGGCCTCAACCTAATCGCCGCCAGCCCCTCGCCCTCCTCT------GCGCTGCAGGCCTTGGGC 6754
          ********* **  ******************* *  ** * *** ************ ************ *  ***   * *   ** **           *   * ** *

Mmus    TTGGGCAAAGGGCAATGGAGCCCCCACCCTACCGGTGTCTGTTCACCCTTTTCTTGTTAAGCCCCCTTTGCTTTTGAAGAACAGAGGG 94921625
Hsa     CCGGGCAAAGGTCAGTGGAGCCCCCACTCGACCTGGGCCTGTTCAGCCTCTTCCTGTTCAGCCTCTTTGGCTTTTGGGGAACCACAGA 6842
          ********* ** ************ * *** * * ******* *** *** **** **** * ** *******  ****    *
```

...

```
Mmus    94915907   GAACATGGTAAGGTGAGCTGGGTTTTACTCTGGGAAAGGTGGGACTGGAAAGTA-----------------GCCTT-----GGGGTTGACCTG 94915837
Hsa        13442   GAGCATGGCAAGGTGAGTGGGCCCCAAGGGGAGTACCATGGGCTGGGGGCAGCATTGGACTGCCAGTGTGACAGTGGGGACGGGGTCCTGGA 13534
                   ** ***** ********  **    *    * *    **    **  ** *              *  *     *** *
```

```
Mmus      AGTGACTGAAAGTTTCCACCCTTTGCTTTCTAGTCAGTGAAAAAGTTCCCCTGTCGCCTGTGTGAGCGCTCCTTTTGCTCTGCCCCAAGCCTGAGGCGCCATGTCAGGGTCAACCATGAG 94915717
Hsa       GCCTGGCTCTGACATCTACCCCTGCTCTCCTAGTCAGTGAAAAAGTTCCCCTGTCGCCTGTGTGAGCGCTCCTTCTGCTCCGCCCCCAGCCTGAGGCGCCATGTCAGAGTTAATCACGAG 13654
                        ** **** *    * ********************************************* ***** ***** ******************** ** ** ** ***

Mmus      GGAATC    94915711
Hsa       GGCATC    13660
          ** ***
```

Supplementary Figure S2. Nucleotide alignment between mouse (Mmus) *Zfp687* and human (Hsa) *ZNF687* gene. The mouse *Zfp687* sequences used for methylation analysis is shown. The numbers correspond to the nucleotide positions according to NCBI accession numbers NC_000069.7 (mouse genomic sequence) and NG_051575.1 (human genomic sequence). CpG dinucleotides are highlighted in green and yellow (conserved with human). Exons are highlighted in grey. The mouse transcription start site (+1) is represented by an arrow.
