# Supplementary material for: Epigenetic Regulation of ZNF687 by miR-142a-3p and DNA Methylation During Osteoblast Differentiation and Mice Bone Development and Aging
Source: Int J Mol Sci. 2025 Feb 27;26(5):2069. doi: 10.3390/ijms26052069 (PMC11899743; doi:10.3390/ijms26052069)
Supplement: Supplementary file 1 [file ijms-26-02069-s001.zip › Supplementary Figure S3.pdf]

-1224

1
CTTGCTTGGACTGGGTGAAGCACTAGGTTCCACTCCGAACACTGCAAAAAGTGAAAATAAAACA

2 3 4 5 6 7 8 9 10
GAAATTTAAAAACCCTTGTCATTTGAAACGGCTGCGGTCGCGGTGGGCGGCGGCGGCGCGGGGC

11 12
CAGGGTAGAGAGCGCGCCAG**GTGGCACACTGGGCCTGCAGATGGATCC**AGCAAAAACCTGAGCC

13 14 15 16
AGCGCAGGTGGGTGAGTGTCAAGGCAATTCGTCTCCCTGGCGACTCCGCCCCTTTTCCCACCTC

17 18 19 20 21
CAAGGGGCGGGGCAGCAAAGCGCGGGAAAGCTCAACGGGAACAGTAATGCCAGCTCCCGTCAGG

22 23 24
GGCGGAGAAAAGG**GAGCTCTCTTAAATGGGAAATAGATCTG**GGCTCCCTCCTTCTCTCGCGTAA

25
GAAGCCTATCAGCATACTACCTTAAGC**TCTCCTTGGTTGTGGCCCAGGCCA**CGCCAGCACTTCC

26 27 28 29 30 31
CAACGTCACCTCCCCCACTTCCGGTCGCAGCGTGTCCCAGGTCTCAGGTCTCGGTGGTTCCGGT

32 33 34 35
TTCTTTTACACTCGGAATCCCAAAGTCTGTCCAGCTCTCCCGCAGAGGGAGGGGCCGTGCGTGG

36 37 38 39 40 41
TGCGTACGCCCAGAGGCAGGCGGACGCCAGAACCGCATCCATCCGGATTATAAAGCAGTTTAGA

42 43 44
CTGCGAGGAACCCCAGGCAATGGTCACCCCGATGACGTAATGTTTGGG**GGTGGCACCTCATTTT**

45 46 47 48 49 50 51
GTGACCCCCAGCACCGCATCCGTTCGAGCGCGGCTTAGCCTGAGGAAACGGCACGAACACTTAA

52 53 54 55 56 57
AACACCCAGGTTCCACACGGAGCCGACGAGGTTCCCCTAGACGCGCCGCCATACAGCTAGAGTT

58 59
CCTAAGTTTGTAGATGTGAGGCACAGTGTCCAGCTTCGAGCGACCAATGGAGAGAGGATGGCTA

60 61 62
CGCTTCTGAGCTAGCTAGTCACCCCTCGCAACCCAAAGCGAAGACTAAGGCTGGCTTCTTGCTA

63 64 65 66 67 68
AGCGACAGCGGCGGCGGCCAATGGCTTTCGGGC**TGCTCAGACAGGGCAGGTGGAC**TCCGGAGCA

GCCAATGGGGAGA**ACCTTCTCCACAGGGCCCAGGCCCTTTG**TCCAATAGTGATGAGAAGACTAT

69 70 71
GAGGGGCCAGTTCCGAGCGGGGTATGGAGGGCCAATCCCATACGGGACTGGGT**GGGGCTCACAG**

TATA-box
72 73 74 75
TTGAATGACAGGTCCATTAGCCAATGGCGAGTATGAAAGCGGGCAGCCCGGACTAGGAGCACGG

+1
76 77 78 79 80 81
AGGGAGCTGAGGGAGAGGGGAGGCCGAAGCGGAGAGAAGCAGGAAGTAGCGGCGGCCGCGGGGA

82 83 84 85 86 87 88 89 90 91
GGGCGGCGGCGGCGGTAGCGGCGGTGGCGGCTGGAACCGGGAGGAGGCCGTAGGGTCTCAGCCC

92 93 94 95 96 97 98
GCACCAGGATCGGAACAAGTAAGCACGCTCGCGGCAAATACCCGCCCCTGGCTCCGCCCCCCGG

TATA-box
99 100 101 102
GGGGGCGGGTAAATACCCCTGCCCCT**CCCCCACTTGGTCAACTACCCCCT**TCTCCGTGCCGCCG

103 104 105 106 107 108

CCCCCTGGGGCGGCTGCTGGCCTGGCCGGGTCGCCGCCTTCTTCTTCGAGGACCACCGGT**GGGA**

109 110

ACTCCAGTTTGCTGGAAGCACGGCCCCCTAGCCTGGTAACGTGGGAGGGGGTTCCTGGACCCCT

111 112 113 114 115

AAGTCCTGGCGCTCTTAGACTGAGAGGCAGTCAGAGTGCGGCCGGGGGACTCGAGGCTGCGGGA

116 117 118 119 120 121

TGTCGCGACCTAAAATGGTGTCCGTGCCGGGCGGTGGGGGCGGGGGAA**TTGGGCCCAGCAGGAG**

122 123 124 125 126

AGGAGCCTGCATCCCGACCGGGTGCTGGTCAGCGTCCGGACCCAGGCAGGACTCAGGCTAGCGC

127 128 129

CTCGATCCTGGCGAGCTCCTTGCAGTCACTCGGGTCTGGGGAGAGCCTC**TGGGGGTCAGAGACA**

130 131 132 133

GGGCAAGCTGCCCGGCGCGCAGAGATGAAGGGGACCAGGTTCGAGTCTGGACTGACCTGGCTGC

134 135 136 137

AGAGCTTAGAATCACGCAGGGACCGCCCCCTCTTGTGTCTCCACTCCCCTCGCGCCACTCCCCA

138 139 140 141

CTGCCGGGGAGTCCTGGCCCCCAATGTGCAGCGCCGCAGATCCTAAGGGCCTGGCCTCACCGAG

142 143

GCCCTCCCTTGAGTTCTTAGCCGGTGAAAGTGAAAGAAGCACCACTCGCCTCAGTTTACCCTTT

144 145 146 147

AGTTTTTAAATGTACTTCTTGTCCCCTACCCGAAACCCCGGACTTACTTTCCGCGCCAGT**CCCT**

148 149 150 151

GGCAGATGGCAGGGACTTAAATGCCGCCCGCTGCCCAGCTTGGCCGCAACCTAATCGCCCCTGG

152 153

CCGTGCTCTGGCCCCTTCCTTTTGCTCTGCGCTGTAGGCCTTGGGCAAAGGGCAATGGAGCCCC

154

CACCCTACCGGTGTCTGTTCACCCTTTTCTTGTTAAGCCC**CCTTTGCTTTTGAAGAACAGAGGG** +1144

...

+6862

GAACATGGTAAGGTGAGCTGGGTTTTACTCTGGGAAAGGTGGGACTGGAAAGTAGCCTTGGGGT

155

TGACCTGAGTGACTGAAAGTTTCCACCCTTTGCTTTCTAGTCAGTGAAAAAGTTCCCCTGTCGC

156 157

CTGTGTGAGCGCTCCTTTTGCTCTGCCCCAAGCCTGAGGCG**CCATGTCAGGGTCAACCATGAGG**

GAATC +7058

Supplementary Figure S3. CpGs dinucleotides in mouse *Zfp687* gene. The *Zfp687* sequence shown corresponds to the region from 94915711 to 94923992, according to NCBI accession number NC_000069.7. CpG dinucleotides are indicated in green and yellow, with the yellow letters representing those conserved with human. Primers sequences are underlined. Exons are highlighted in grey. Dashed boxes indicate TATA-box sequences. The transcription start sites are represented by arrows. CpGs dinucleotides are numbered (1 to 157) from 5′ to 3′ direction.
