# Supplementary material for: Epigenetic Regulation of ZNF687 by miR-142a-3p and DNA Methylation During Osteoblast Differentiation and Mice Bone Development and Aging
Source: Int J Mol Sci. 2025 Feb 27;26(5):2069. doi: 10.3390/ijms26052069 (PMC11899743; doi:10.3390/ijms26052069)
Supplement: Supplementary file 1 [file ijms-26-02069-s001.zip › Supplementary Figure S4.pdf]

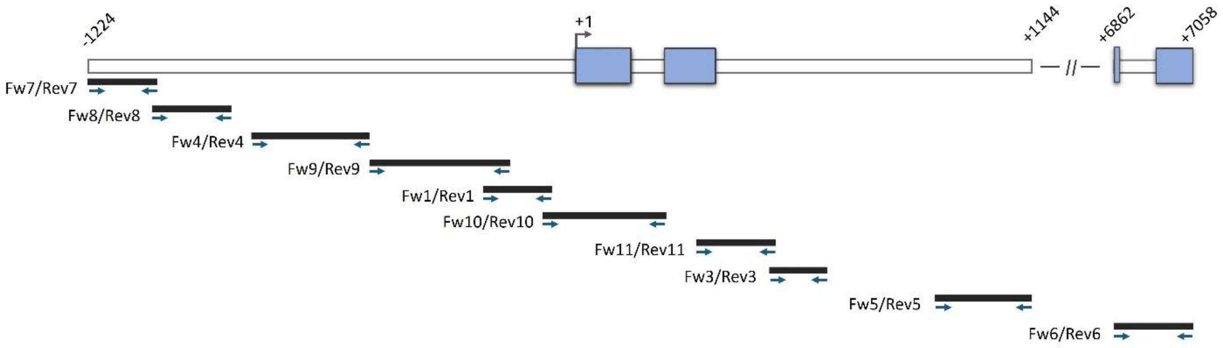

Supplementary Figure S4. Schematic representation of bisulfite primers annealing in *Zfp687* and ten resultant amplicons. *Zfp687* exons and introns are represented by blue boxes and horizontal lines, respectively**.** Numeric nucleotide positions are indicated according to the transcription start site (+1). Sequence from +1144 to +6862 is not represented. Blue arrows indicate the forward (Fw) and reverse (Rev) primers and black bars the corresponding amplicon.
