# Supplementary material for: Epigenetic Regulation of ZNF687 by miR-142a-3p and DNA Methylation During Osteoblast Differentiation and Mice Bone Development and Aging
Source: Int J Mol Sci. 2025 Feb 27;26(5):2069. doi: 10.3390/ijms26052069 (PMC11899743; doi:10.3390/ijms26052069)
Supplement: Supplementary file 1 [file ijms-26-02069-s001.zip › Supplementary Table S1.pdf]

Supplementary Table S1. List of the primers used in this work. Underlined sequences indicate restriction site for endonucleases cited in primer name. Fw: forward primer; Rev: reverse primer.

Name	Sequence (5' to 3')
**Gene expression primers**	
Mmus_Znf687_qPCR_Fw2	AAGGAACATGGTAAGTCAGT
Mmus_Znf687_qPCR_Rev2	GACCTGAACATGCTTCTCCA
Mmus_Sp7_qPCR_01Fw	TCCTATGCTCCGACCTCCTCAACTTTT
Mmus_Sp7_qPCR_01Rev	GGAAGCAGAAAGATTAGATGGCAACGAG
Mmus_Alpl_qPCR_Fw2	CTGGGAGATGGTATGGGCGT
Mmus_Alpl_qPCR_Rev2	CTGAGCGTTGGTGTTATATGTCTTGG
Mmus_Coll1a_qPCR_Fw3	TGACAAGGGTGAGACAGGCGA
Mmus_Coll1a_qPCR_Rev3	CACCAGGAGAACCAGGAGAACCAG
Mmus_Bglap_qPCR_Fw1	CCTCACAGATGCCAAGCCCA
Mmus_Bglap_qPCR_Rev1	GCGGTCTTCAAGCCATACTGGTC
Mmus_Gapdh_Fw2	AGGTCGGTGTGAACGGATTTG
Mmus_Gapdh_Rev2	CGTGAGTGGAGTCATACTGGAAC
**miRNAs expression primers**	
Mmus-miR-124-3p_Fw	TAAGGCACGCGGTGAATGC
Mmus-miR-142a-3p_Fw	GTAGTGTTTCCTACTTTATGGA
Mmus-miR-122b-5p_Fw	TTTAGTGTGATAATGGCGTTTG
Mmus_U6_Fw	AGGATGACACGCAAATTCGTG
**Bisulfite-converted DNA amplification primers**	
Mmus_Znf687_BSP_Fw1	TGTTTAGATAGGGTAGGTGGAT
Mmus_Znf687_BSP_Rev1	AAACCTATCATTCAACTATAAACCCC

Mmus_Znf687_BSP_Fw3	GGTTTAGTAGGAGAGGAGTTTGTA
Mmus_Znf687_BSP_Rev3	ACTTACCCTATCTCTAACCCCCA
Mmus_Znf687_BSP_Fw4	TTTTTTTGGTTGTGGTTTAGGTTA
Mmus_Znf687_BSP_Rev4	AATCACAAAATAAAATACCACCCC
Mmus_Znf687_BSP_Fw5	TTTTGGTAGATGGTAGGGAT
Mmus_Znf687_BSP_Rev5	CCCTCTATTCTTCAAAAACAAAAA
Mmus_Znf687_BSP_Fw6	GAATATGGTAAGGTGAGTTGGGTTTT
Mmus_Znf687_BSP_Rev6	AATTCCCTCATAATTAACCCTAACATAA
Mmus_Znf687_BSP_Fw7	TTTGTTTGGATTGGGTGAAGTATT
Mmus_Znf687_BSP_Rev7	AAATCCATCTACAAACCCAATATACCAC
Mmus_Znf687_BSP_Fw8	GTTTGTAGATGGATTTAGTAAAAATTTG
Mmus_Znf687_BSP_Rev8	CAAATCTATTTCCCATTTAAAAAAACTC
Mmus_Znf687_BSP_Fw9	GTGGTATTTTATTTTGTGATTTTTAGTA
Mmus_Znf687_BSP_Rev9	CAAAAAACCTAAACCCTATAAAAAAAAT
Mmus_Znf687_BSP_Fw10	GGGTTTATAGTTGAATGATAGG
Mmus_Znf687_BSP_Rev10	AAAAAATAATTAACCAAATAAAAA
Mmus_Znf687_BSP_Fw11	GGGAATTTTAGTTTGTTGGAAGTA
Mmus_Znf687_BSP_Rev11	CTCCTCTCCTACTAAACCCAA
***Znf687* 3'UTR cloning primers**	
Mmus_Znf687_3UTR_Fw_SpeI	GGACTAGTGGGACTTGGGTTTCACTGCT
Mmus_Znf687_3UTR_Rev_HindIII	CTAAAGCTTCCATCTGTCTTCGTCAATACTGCC
***Znf687* 3'UTR mutagenesis primers**	
Mmus_Znf687_3UTR_mut_Fw	CTTCTTTTTCCTTTCTGAATCCAGCGTTGATGCTCCTGCTGCAGACTCCCAG
Mmus_Znf687_3UTR_mut_Rev	CTGGGAGTCTGCAGCAGGAGCATCAACGCTGGATTCAGAAAGGAAAAAGAAG

Sequencing primers	
SP6	ATTTAGGTGACACTATAG
T7	TAATACGACTCACTATAGGG
M13	TGTAAAACGACGGCCAGT
